# The NLRP3 inflammasome as a bridge between myocardial infarction and depression: mechanisms, bidirectional link, and therapeutic potential

**DOI:** 10.3389/fcvm.2026.1924945

**Published:** 2026-10-08

**Authors:** Pingping Zhou, Yuan Ma, Chen Hou, Cuicui Chen, Shuang Deng

**Affiliations:** 1 Tianhua College, Shanghai Normal University, Shanghai, China; 2 Department of Rehabilitation Medicine, The Second Affiliated Hospital of Anhui Medical University, Hefei, China

**Keywords:** depression, gut-brain axis, heart-brain comorbidity, myocardial infarction, NLRP3 inflammasome, oxidative stress, therapeutic target, treatment resistance

## Abstract

Myocardial infarction (MI) and depression frequently co-occur and form a bidirectional relationship that worsens patient outcomes, with inflammation serving as a core mechanism linking these two disorders. The NLRP3 inflammasome, a key component of the innate immune system, plays a pivotal role in sterile inflammatory responses. This review systematically summarizes the structural characteristics, activation mechanisms, and pathophysiological functions of the NLRP3 inflammasome, with a particular focus on its involvement in the pathogenesis of MI and depression and the bidirectional relationship between the two diseases. We comprehensively elaborate on the role of the NLRP3 inflammasome as a molecular bridge between MI and depression, exploring mechanisms including oxidative stress and mitochondrial dysfunction, ATP-P2X7 signaling and calcium pathways, peripheral and central inflammation, sympathetic and vagus nerve pathways, the gut microbiota-gut-brain axis, neurotransmitter and neurotrophic metabolic dysregulation, VEGF-mediated vascular permeability, metabolic disturbances, and neuroendocrine dysfunction. We also discuss the clinical implications of NLRP3-related molecules as biomarkers for predicting treatment responses and analyze the role of the NLRP3 inflammasome in treatment sensitivity and resistance within heart-brain comorbidity. Finally, we summarize current therapeutic strategies targeting the NLRP3 inflammasome and propose future research directions. We propose that NLRP3 inflammasome activation is a common upstream driver of both cardiac remodeling and depressive neuroinflammation, and that its persistence explains why standard therapies often fail in comorbid patients. Targeting NLRP3 may therefore break the self-sustaining inflammatory loop that perpetuates the comorbidity. Targeting the NLRP3 inflammasome may represent a novel strategy for breaking the vicious cycle between MI and depression, offering potential benefits for both cardiovascular and mental health. Importantly, direct evidence for NLRP3 as a causal mediator in comorbidity-specific systems remains limited; we therefore present this as an integrative framework that synthesizes parallel pathways and identifies critical knowledge gaps.

## 1 Introduction

Myocardial infarction (MI) and depression are two of the leading causes of death and disability worldwide (1, 2). Cardiovascular diseases, predominantly ischemic heart disease, account for approximately 17.9 million deaths annually (32% of all global deaths), while depression affects an estimated 280 million people (3.8% of the global population) (1, 3, 4). These two disorders do not exist independently, but rather exhibit significant comorbidity. Epidemiological studies have shown that patients with depression have an approximately 1.3-fold higher risk of developing MI compared with the general population (5). A meta-analysis reported a pooled prevalence of post-MI depression of 23.58%, with the prevalence being 19.46% within the first 3 months after infarction and 14.87% thereafter (5, 6). This decline over time suggests that psychological distress is most pronounced in the acute post-infarction period and may gradually subside as patients physically recover, although the pooled estimate exceeds each subgroup estimate because the overall prevalence reflects the combined proportion across all time points, with the majority of studies reporting early post-MI assessment.

Inflammatory response has been identified as a core mechanism linking MI and depression (7, 8). In MI, ischemic necrotic tissue releases abundant damage-associated molecular patterns, activating the innate immune system and triggering sterile inflammatory responses, which exacerbate myocardial injury, ventricular remodeling, and heart failure progression (7, 8). In depression, chronic stress induces persistent elevation of peripheral and central inflammatory cytokines, leading to microglial activation, neurotransmitter metabolism dysregulation, impaired synaptic plasticity, and suppressed neurogenesis (9–11). Notably, patients with MI frequently exhibit elevated systemic inflammatory markers, including C-reactive protein, interleukin-6, and tumor necrosis factor-alpha, and these inflammatory factors are also significantly upregulated in patients with depression (12–14).

The NLRP3 inflammasome is a core component of the innate immune system and serves as a sensor for various endogenous and exogenous danger signals, playing a pivotal role in sterile inflammatory responses (15). Among the NOD-like receptor family, NLRP3 is distinguished by its broad activation spectrum and its ability to respond to diverse structurally unrelated stimuli, including ATP, cholesterol crystals, and mitochondrial reactive oxygen species, which are all relevant to both MI and depression pathophysiology (16–18). The NLRP3 inflammasome is composed of the NLRP3 receptor protein, the adaptor protein ASC, and the effector protein pro-caspase-1 (16). Its activation requires dual regulation by priming and activating signals (15). In the cardiovascular system, NLRP3 is expressed in cardiomyocytes, endothelial cells, and infiltrating macrophages, where it mediates sterile inflammation following ischemic injury. In the central nervous system, NLRP3 is predominantly expressed in microglia, with lower levels in neurons and astrocytes, and its activation drives neuroinflammation that contributes to depressive-like behaviors (11). Upon activation, the NLRP3 inflammasome promotes the maturation and secretion of interleukin-1beta (IL-1β) and IL-18, induces pyroptosis, and amplifies inflammatory cascades (17).

The mechanistic link between MI and depression is multifaceted. MI triggers systemic inflammation that affects the central nervous system through cytokine signaling, vagal afferent transmission, and blood-brain barrier disruption (7, 8). Conversely, depression involves chronic low-grade inflammatory response that promotes atherosclerosis and endothelial dysfunction. This bidirectional relationship suggests that shared inflammatory mechanisms underpin the comorbidity. Among inflammatory pathways, the NLRP3 inflammasome occupies a central position. Unlike other mediators, NLRP3 directly senses diverse damage signals and integrates them into a common inflammatory output. Its activation produces IL-1β and IL-18, cytokines implicated in both cardiovascular pathology and depressive disorders. NLRP3 is expressed in both peripheral immune cells and central microglia, positioning it at the interface of peripheral and central inflammation (15). This dual distribution and broad activation spectrum distinguish NLRP3 from other inflammasome family members. These properties make NLRP3 a compelling molecular bridge for understanding heart-brain comorbidity and a promising therapeutic target.

Current pharmacological management of post-MI depression relies primarily on selective serotonin reuptake inhibitors and other antidepressants, yet a substantial proportion of patients fail to respond adequately to these agents. The mechanisms underlying this treatment resistance remain poorly understood. The role of the NLRP3 inflammasome in heart-brain comorbidity has received extensive attention, particularly regarding treatment sensitivity and resistance. However, the spatiotemporal dynamics of NLRP3 activation across different pathological stages of MI and depression comorbidity remain poorly understood. Based on the above background, this review summarizes the molecular mechanisms of the NLRP3 inflammasome in the bidirectional relationship between MI and depression, discusses the therapeutic strategies targeting the NLRP3 inflammasome and their potential in improving comorbidity treatment resistance, and proposes current research limitations and future directions, aiming to provide a theoretical basis for precision treatment of heart-brain comorbidity. This review advances the working hypothesis that NLRP3 inflammasome activation constitutes a mechanistic bridge between MI and depression, while critically evaluating the strength of evidence supporting this model and identifying key knowledge gaps, particularly the need for causal validation in comorbidity-specific experimental systems. This review integrates the NLRP3 inflammasome into a unified mechanistic framework linking MI and depression, distinguishes direct comorbidity-specific evidence from indirect single-disease evidence, proposes a hierarchical spatiotemporal model of NLRP3 activation across acute, subacute, and chronic phases, and identifies key knowledge gaps and the evidence needed to change clinical practice. It therefore positions NLRP3 as a potential upstream driver and therapeutic target in MI–depression comorbidity, rather than merely a downstream inflammatory marker.

This review was conducted by systematically searching PubMed, Web of Science, Embase, and the Cochrane Library for literature published from January 2000 to September 2026.The search combined terms related to the NLRP3 inflammasome (“NLRP3 inflammasome”, “NLRP3”, “inflammasome”, “IL-1β”, “IL-18”, “pyroptosis”, “caspase-1”), myocardial infarction (“myocardial infarction”, “MI”, “acute coronary syndrome”, “ischemia–reperfusion injury”, “cardiac remodeling”, “heart failure”), and depression (“depression”, “depressive disorder”, “major depressive disorder”, “depressive-like behavior”, “post-MI depression”, “heart–brain comorbidity”, “neuroinflammation”). Additional keywords included “oxidative stress”, “mitochondrial dysfunction”, “ATP–P2X7”, “gut–brain axis”, “vagus nerve”, “BDNF”, “IDO–kynurenine pathway”, “VEGF”, “metabolic dysregulation”, and “treatment resistance.” Both original research articles and reviews were considered. Studies were included if they investigated the role of the NLRP3 inflammasome in myocardial infarction, depression, or their comorbidity, or if they provided mechanistic evidence relevant to heart–brain crosstalk. Articles not written in English, duplicate publications, and studies without full-text access were excluded. Reference lists of included articles were also screened manually to identify additional relevant studies. This review was not registered, and no formal meta-analysis was performed because of the heterogeneity of study designs and outcome measures.

## 2 The structure and pathophysiological function of the NLRP3 inflammasome

### 2.1 Family subtypes, structure, classification, and physiological and biochemical characteristics

The NLRP3 inflammasome is one of the most extensively studied members of the NOD-like receptor family. The NLR family comprises more than 20 members. Among them, NLRP1, NLRP3, NLRC4, and AIM2 have been identified as receptor proteins capable of forming functional inflammasomes (18, 19). Compared with other NLR family members, NLRP3 can be activated by a wide variety of structurally diverse stimuli, including pathogen-associated molecular patterns, damage-associated molecular patterns, and environmental irritants (20). This broad activation spectrum underlies its central role in various sterile inflammatory diseases. NLRP3 is distinguished from other NLR family members by its exceptionally broad ligand specificity. Structurally, this property is attributed to the leucine-rich repeat domain at the C-terminus, which undergoes conformational changes upon ligand binding, and the centrally located NACHT domain, which mediates ATP-dependent oligomerization (21, 22). Unlike NLRP1 and NLRC4, which respond to restricted sets of stimuli, NLRP3 can be activated by diverse structurally unrelated agonists, including extracellular ATP, pore-forming toxins, crystalline substances, and mitochondrial ROS. This broad ligand specificity suggests that NLRP3 senses common cellular stress signals rather than specific ligands (20, 24). Importantly, while most NLRP3 research has been conducted in human and murine systems, the NLRP3 inflammasome components are evolutionarily conserved across mammals, with orthologs identified in other species, though their ligand specificities and activation mechanisms may differ (18, 19).

The NLRP3 inflammasome is a multi-protein complex composed of three core components. The first is the sensor protein NLRP3, whose molecular structure contains three functional domains (21). In addition, the C-terminal leucine-rich repeat domain is responsible for ligand recognition and maintaining self-inhibition (21). The central nucleotide-binding and oligomerization domain possesses ATPase activity and is critical for NLRP3 activation and oligomerization (22). And the N-terminal pyrin domain mediates downstream signal transduction. The second component is the adaptor protein ASC, which contains a pyrin domain and a caspase recruitment domain, bridging NLRP3 and downstream effector proteins (23). The third component is the effector protein pro-caspase-1, which upon activation mediates downstream inflammatory responses (23). These three components sequentially assemble into a functional inflammasome complex through homotypic interactions of pyrin-pyrin domains and pyrin-caspase recruitment domains.

Activation of the NLRP3 inflammasome requires dual signal regulation. The first signal, known as the priming signal, is triggered by pathogen-associated molecular patterns or damage-associated molecular patterns via Toll-like receptors or cytokine receptors, activating the NF-κB signaling pathway and subsequently upregulating the transcriptional expression of NLRP3, pro-IL-1β, and pro-IL-18 (24). The second signal, known as the activation signal, is provided by various stimuli including extracellular ATP, pore-forming toxins, crystals, or particulates. These stimuli trigger intracellular K⁺ efflux, lysosomal cathepsin B leakage, mitochondrial reactive oxygen species production, and mitochondrial DNA release. Consequently, these events culminate in the assembly and activation of the NLRP3 inflammasome (25). In addition to this canonical two-signal pathway, non-canonical and alternative activation pathways also participate in NLRP3 inflammasome regulation under specific cell types and stimulatory conditions. The non-canonical pathway involves caspase-11 (in mice) or caspase-4/5 (in humans) directly binding intracellular lipopolysaccharide, leading to caspase-1-independent pyroptosis and K⁺ efflux that secondarily activates NLRP3 (25). The alternative pathway, primarily described in human monocytes, bypasses the requirement for a separate activation signal, as TLR4 engagement alone can trigger NLRP3 activation through a pathway involving receptor-interacting protein kinase 1 (RIPK1) and caspase-8, without requiring K⁺ efflux (25).

The tissue distribution of NLRP3 exhibits marked tissue specificity. Studies have shown that NLRP3 mRNA is most highly expressed in the kidney and inguinal lymph nodes (26). Immunohistochemical detection has revealed that NLRP3 protein is expressed in a wide range of tissues and cell types, including cardiomyocytes, cerebral cortical neurons, epithelial reticular cells of the spleen and thymus, lymphocytes in inguinal lymph nodes, alveolar macrophages, renal tubular cells, and hepatic sinusoidal endothelial cells (26, 27). Furthermore, NLRP3 is also expressed in B lymphocytes and T lymphocytes, mucosal epithelial cells of the oral cavity and reproductive tract, keratinocytes, and chondrocytes (27). This broad tissue distribution provides a structural basis for its role in various organ system diseases.

### 2.2 Physiological homeostasis maintenance and pathological hyperactivation

Under physiological conditions, the NLRP3 inflammasome is maintained at a basal level of activation and participates in tissue repair and homeostasis. It promotes extracellular matrix remodeling and angiogenesis following tissue injury, and may contribute to immune surveillance by recognizing abnormally proliferating or senescent cells, thereby maintaining the immunological balance of the tissue microenvironment (19, 28).

In response to pathogenic microbial infection or other danger signals, NLRP3 can act as a pattern recognition receptor and become activated to initiate host defense (24). This infection-triggered activation represents a controlled and beneficial response under physiological conditions, distinct from pathological hyperactivation. Persistent or excessively strong stimuli lead to aberrant assembly and activation of the NLRP3 inflammasome, triggering excessive release of IL-1β and IL-18 and inducing a potent inflammatory cascade (17). Hyperactivated caspase-1 cleaves gasdermin D, generating an N-terminal fragment with pore-forming activity, which forms non-selective pores in the cell membrane, leading to cell swelling, membrane rupture, and leakage of intracellular contents, a process known as pyroptosis (17, 24). Pyroptosis not only results in cell death but also releases a large quantity of pro-inflammatory mediators, further amplifying the local inflammatory response (29).

It is important to note that NLRP3 activation is not synonymous with pyroptosis. Under conditions of subthreshold or moderate NLRP3 activation, caspase-1 can process pro-IL-1β and pro-IL-18 into their mature forms without inducing gasdermin D cleavage and subsequent cell death. These cytokines can be secreted through alternative pathways, including gasdermin D-independent secretory mechanisms and extracellular vesicles (17). This uncoupling of cytokine maturation from pyroptosis is particularly relevant in chronic inflammatory states, where sustained low-grade IL-1β and IL-18 release contributes to tissue remodeling and disease progression without overt cell loss (29).

Beyond caspase-1-dependent cytokine processing, NLRP3 exerts caspase-1-independent functions that are increasingly recognized in both cardiovascular and neurological contexts. NLRP3 can regulate mitochondrial homeostasis, modulate NF-κB signaling through its interaction with ASC, and influence gene transcription independently of inflammasome formation (15). NLRP3 can also activate caspase-8 to induce apoptosis or interact with RIPK1 to promote necroptosis, representing alternative cell death pathways that operate independently of caspase-1 and gasdermin D (17, 29–31). These caspase-1-independent functions expand the repertoire of NLRP3-mediated biology beyond classical inflammasome activation and have important implications for understanding its role in complex disease states such as MI-depression comorbidity, where pyroptosis may not be the dominant pathological mechanism.

Hyperactivation of the NLRP3 inflammasome is closely associated with a wide range of inflammatory diseases, including disorders of the nervous system, cardiovascular system, respiratory system, digestive system, urinary system, endocrine system, and rheumatic immune system. The specific roles of NLRP3 in MI and depression, including its activation mechanisms and downstream pathological consequences in these two conditions, are discussed in detail.

## 3 Epidemiological evidence for the bidirectional relationship between depression and MI

Epidemiological studies have established a bidirectional relationship between depression and MI. This section reviews the epidemiological evidence for both directions, with detailed mechanistic discussions provided in Section 5.

### 3.1 Depression as a risk factor for MI

Depression is not only a psychological burden but also an independent risk factor for MI. A meta-analysis, which included 26 studies comprising a total of 1,957,621 individuals, demonstrated that patients with depression had a hazard ratio (HR) of 1.28 for developing MI (5). Meanwhile, depression was also significantly positively associated with cardiovascular disease mortality and all-cause mortality. A systematic review and meta-analysis by Gupta et al., encompassing 25 studies with over 22 million participants, further confirmed that depressive disorder is significantly associated with an increased risk of acute coronary syndrome (32). Although acute coronary syndrome represents a broader category than MI, the strong association observed in these studies supports the role of depression in precipitating acute ischemic events, including MI. Moreover, anxiety disorder, post-traumatic stress disorder, and sleep disturbances have also been identified as independent risk factors for acute coronary syndrome (32).

Inflammatory mechanisms have been proposed as key mediators of these epidemiological associations. Chronic stress and depression are associated with sustained elevations of pro-inflammatory cytokines and sympathetic nervous system overactivation, which can promote atherosclerosis progression, endothelial dysfunction, and plaque instability, processes that ultimately increase the risk of MI (7, 8). Experimental studies have shown that chronic stress can prime and activate the NLRP3 inflammasome in both peripheral immune cells and central microglia, suggesting a plausible mechanistic pathway through which depressive pathology may increase cardiovascular vulnerability (11, 24). However, direct clinical evidence linking pre-existing depression to NLRP3 inflammasome activation in the context of subsequent MI remains limited and warrants further investigation.

### 3.2 MI as a trigger for depression

MI not only causes structural damage to the heart but also significantly increases the risk of developing depression. A meta-analysis by Chong et al., which included 25 studies using structured clinical interviews for diagnosis, found that the pooled prevalence of post-MI depression was 23.58%, with a prevalence of 19.46% within the first 3 months after infarction and 14.87% thereafter, indicating that the early post-infarction period is a critical window for depression intervention (6, 33, 34). Another meta-analysis encompassing 13 studies reported a higher pooled prevalence estimate of 34.1%, further confirming the substantial burden of post-MI depression (34). Further risk factor analysis revealed that female sex, comorbid hypertension, hyperlipidemia or diabetes mellitus, smoking, unmarried status, and a history of depression significantly increased the risk of post-infarction depression (34).

To investigate the involvement of the NLRP3 inflammasome in post-MI depression, Baysak et al. conducted a clinical study enrolling 68 patients with acute ST-segment elevation MI and 20 healthy controls, using the DSM-5 structured clinical interview to assess depressive status (33). The results showed that plasma NLRP3 levels in MI patients were significantly higher than those in healthy controls. Logistic regression analysis indicated that plasma NLRP3 levels were independently associated with the occurrence of post-MI depression, with an odds ratio of 1.72 (33). This finding provides preliminary clinical evidence of an association between circulating NLRP3 levels and post-MI depression, independent of several measured covariates. However, the observational nature of this study and the absence of formal mediation analysis preclude causal or mechanistic inferences. The reported odds ratio reflects a statistical association after covariate adjustment rather than evidence of a causal pathway, and the original study does not report the unit of NLRP3 increase or the specific covariates adjusted for, limiting interpretability. Therefore, this finding should be interpreted strictly as an association and should not be overinterpreted as evidence that NLRP3 drives the development of depression. Consistent with this cautious interpretation, we present this clinical observation as supportive correlational evidence that complements the substantial preclinical mechanistic data, rather than as proof of a causal relationship. Prospective studies with formal mediation frameworks and longitudinal biomarker assessments are warranted to establish temporal and causal relationships.

The concept of vascular depression provides a broader framework for understanding post-MI depressive disorders. Vascular depression was proposed over two decades ago as a distinct subtype of depression primarily observed in patients with stroke and cerebral small-vessel disease and is characterized by white matter hyperintensities (34). This concept is particularly relevant to post-MI depression, as myocardial infarction shares common vascular risk factors with cerebrovascular disease, and the systemic inflammatory response following MI may compromise blood-brain barrier integrity and contribute to cerebral microvascular injury (34, 35). Recent comprehensive reviews have categorized the pathophysiological mechanisms of vascular depression into three groups, including macroscopic mechanisms such as vascular aging, cerebral hypoperfusion, blood-brain barrier disruption, and neural circuit dysfunction, microscopic mechanisms involving the inflammatory response, hypothalamic-pituitary-adrenal axis dysregulation, impaired monoamine synthesis, and mitochondrial dysfunction, and undetermined mechanisms such as microbiota-gut-brain axis dysbiosis (34). These mechanisms overlap substantially with the NLRP3 inflammasome-mediated pathways discussed in this review, as NLRP3 activation contributes to both vascular inflammation and neuroinflammation. Notably, patients with vascular depression often exhibit resistance to conventional antidepressants, a feature that parallels the treatment resistance observed in post-MI depression and further supports the relevance of this conceptual framework to the heart-brain comorbidity discussed here.

### 3.3 Factors affecting comorbidities

The bidirectional association between MI and depression is not a simple causal relationship but rather the result of the combined effects of multiple external environmental factors. Gender, age, lifestyle, social background, comorbidities, and genetic factors all play important roles in the development of the co-morbidity. From a demographic perspective, women are more likely to develop depressive symptoms after MI (36). A study of 944 patients with acute coronary syndrome found that the incidence of depressive symptoms in women was as high as 45%, while in men it was only 35% (36). Further analysis confirmed that women are an independent predictor of depressive symptoms, with a risk increase of approximately 60% compared to men (36). This gender difference suggests that psychological intervention after MI should particularly focus on female patients. Moreover, experimental data indicate that quitting smoking and controlling blood sugar are core strategies for cardiovascular health and may be effective ways to reduce the burden of depression-MI co-morbidity (37).

The routine treatment drugs after MI may have a bidirectional impact on the risk of depression. The association between beta-blockers and depression is controversial. Lipophilic beta-blockers, such as propranolol, can easily penetrate the blood-brain barrier and may interfere with neurotransmitter signal transmission, thereby inducing depression (38, 39). The anti-inflammatory properties of statins may have an antidepressant effect, but some studies report that they are associated with an increased risk of depression (40). The association between antiplatelet drugs and depression is also inconsistent (40, 41). Comorbid patients often need to take both antidepressant drugs and cardiovascular drugs simultaneously, and antidepressant drugs themselves may also have adverse effects on the cardiovascular system. A pharmacovigilance analysis covering the period from 2004 to 2023 and involving 37 commonly used antidepressants showed that these drugs were associated with 8 types of cardiovascular adverse events, including QT interval prolongation, hypertension, arrhythmia, and cardiomyopathy (42). The risks of different types of antidepressants were significantly different, among which monoamine oxidase inhibitors had the highest risks in QT interval prolongation, hypertension, and arrhythmia (42).

Beyond the effects of cardiovascular medications on depression risk, the reciprocal influence of antidepressant treatment on cardiac outcomes in patients with myocardial infarction has also received considerable research attention. A recent systematic review and meta-analysis found that antidepressant treatment significantly reduced depression severity in post-MI patients without increasing the risk of adverse cardiac events or all-cause mortality (8). The choice of antidepressant class appears to be an important determinant of cardiovascular safety. Tricyclic antidepressants have been associated with increased heart rate, reduced heart rate variability, and QT interval prolongation, and some evidence suggests they may increase the risk of myocardial infarction (2, 6). Conversely, selective serotonin reuptake inhibitors are generally considered to have a more favorable cardiovascular safety profile and are often recommended as the first-line pharmacological option for treating depression in patients with ischemic heart disease (3, 6). Among SSRIs, sertraline has been most extensively studied in post-MI populations, with evidence supporting its efficacy in improving depressive symptoms without adversely affecting cardiac function or increasing cardiovascular events (6, 8). However, safety considerations including QT interval prolongation and bleeding risk warrant careful evaluation when prescribing SSRIs to patients with cardiovascular disease (12). Collectively, these observations underscore the importance of individualized antidepressant selection that balances psychiatric efficacy with cardiovascular safety in post-MI patients.

## 4 Evidence for NLRP3 involvement in treatment sensitivity and resistance in heart-brain comorbidity: from preclinical mechanisms to clinical observations

Before examining the detailed mechanistic pathways in Section 5, it is instructive to consider the clinical problem of treatment resistance in post-MI depression. Treatment resistance in post-MI depression affects 20%–30% of patients and is associated with worse cardiovascular outcomes, yet its mechanisms remain unclear (43, 44). Emerging evidence suggests that NLRP3-driven inflammatory cascade may play a critical role. The mechanistic basis for this treatment resistance is discussed in detail in Section 5, following a comprehensive review of the underlying pathophysiological pathways.

Persistent NLRP3 activation sustains systemic and central inflammation, which may interfere with antidepressant efficacy by impairing serotonin synthesis, reducing BDNF expression, and promoting glutamatergic excitotoxicity (11). Concurrently, NLRP3-mediated vascular inflammation may attenuate the cardioprotective effects of standard post-MI medications in depressed patients. The following subsections synthesize evidence linking NLRP3 to treatment responses, examining whether NLRP3-driven inflammation explains antidepressant failure and reduced cardioprotective efficacy in comorbid patients, and whether targeting this pathway could overcome resistance.

Clinically, the response of post-MI depression patients to antidepressant medications exhibits significant heterogeneity. Epidemiological data indicate that approximately 30%–40% of patients are sensitive to conventional antidepressant therapy, with depressive symptoms markedly alleviated within 4–6 weeks, whereas 20%–30% of patients manifest treatment resistance, showing no significant improvement even after adequate dose and duration of treatment (43). Treatment resistance in post-MI depression has a substantial impact on patient prognosis. A sub-analysis of the MIND-IT trial by de Jonge et al. demonstrated that post-MI patients who failed to respond to antidepressant treatment had a 24-week cardiovascular event rate of 25.6%, which was higher than that of treatment responders and even higher than that of the untreated control group (44). A secondary analysis of the ENRICHD study further confirmed that treatment-resistant depression accounted for 13.4% of post-MI depression patients and was an independent predictor of mortality. Collectively, these clinical findings indicate that treatment resistance in post-MI depression is not merely a psychiatric treatment failure but also an independent marker of poor cardiovascular prognosis (44).

The expression of the NLRP3 inflammasome in the brain exhibits marked region-specificity, providing important clues for understanding the neuroanatomical basis of treatment resistance in MI-depression comorbidity. Studies have demonstrated that vortioxetine can specifically reverse lipopolysaccharide-induced upregulation of NLRP3 and ASC in memory-related brain regions, such as the dorsal hippocampus, an effect potentially mediated by modulating NEK7 expression (45). Notably, the regulatory effect of vortioxetine on NLRP3 is time-dependent and brain region-specific, acting primarily on the dorsal hippocampus while exerting no significant effects on other inflammasomes such as NLRP1, NLRC4, and AIM2, suggesting that vortioxetine specifically regulates the NLRP3 inflammasome signaling pathway (45). Furthermore, Ozdemir Unal et al. confirmed that vortioxetine significantly reduces the expression levels of NLRP3, IL-1β, and IL-18 in the prefrontal cortex of rats subjected to chronic unpredictable stress and inhibits the expression of microglial activation markers CD68 and LCA (46).

The dorsal raphe nucleus, as the primary source of cerebral serotonin, is a crucial brain region connecting emotional regulation and cardiovascular function (47). Chronic stress significantly increases NLRP3 expression in microglia and neurons of the dorsal raphe nucleus, leading to dysfunction of the serotonergic system, which may represent a key mechanism underlying treatment resistance to selective serotonin reuptake inhibitors in post-MI depression patients (48). Subsequent administration of the NLRP3 inhibitor MCC950 or conditional knockout of NLRP3 in the dorsal raphe nucleus significantly ameliorated the depressive phenotype (47, 49).

From the perspective of treatment sensitivity and resistance, the NLRP3 inflammasome plays a pivotal role as a molecular switch. Postmortem brain studies have confirmed significantly elevated NLRP3 expression in the prefrontal cortex of patients with major depressive disorder who died by suicide, indicating that persistent NLRP3 activation is closely associated with treatment resistance (50). The impact of routine post-MI medications on antidepressant treatment sensitivity reflects another dimension of heart-brain bidirectional regulation. Lipophilic beta-blockers, such as propranolol and metoprolol, readily cross the blood-brain barrier and may affect central nervous system function, whereas hydrophilic beta-blockers, such as atenolol, have poor penetration (51). Preclinical studies have further elucidated the upstream regulatory mechanisms of treatment resistance. Total paeony glycoside (TPG) inhibits NLRP3 inflammasome activation by activating autophagy, restoring mitochondrial function, and reducing reactive oxygen species accumulation, thereby alleviating neuroinflammation and depressive-like behavior (52). TPG significantly reduces the expression of NLRP3, pro-caspase-1, caspase-1, IL-1β, and the pyroptosis-related protein GSDMD-N, while promoting mitophagy to clear damaged mitochondria. Notably, treatment with the autophagy inhibitor 3-methyladenine attenuates the regulatory effects of TPG, whereas the caspase-1 inhibitor VX-765 exhibits a synergistic therapeutic effect with TPG (52). Furthermore, promoting mitophagy, increasing the clearance of damaged mitochondria, and reducing mitochondrial reactive oxygen species accumulation effectively inhibit NLRP3 inflammasome activation in microglia, thereby exerting antidepressant and neuroprotective effects (53).

Collectively, these observations suggest that NLRP3 inflammasome activation may represent a common pathway through which inflammation undermines both antidepressant and cardioprotective efficacy in post-MI depression. Persistent NLRP3-driven IL-1β and IL-18 production sustains neuroinflammation that impairs serotonergic and glutamatergic neurotransmission, potentially explaining reduced responsiveness to SSRIs. Simultaneously, NLRP3-mediated vascular inflammatory response and sympathetic overactivation may limit the efficacy of cardioprotective drugs in depressed patients. This dual role positions NLRP3 as a promising target for overcoming treatment resistance specifically in comorbid patients. However, direct clinical evidence linking NLRP3 inhibition to improved treatment responses in this population remains lacking. Future trials should prospectively evaluate whether NLRP3-targeted therapies enhance both antidepressant and cardiovascular outcomes in treatment-resistant post-MI depression patients, with baseline NLRP3 levels as potential biomarkers for patient stratification.

## 5 NLRP3 in the pathogenesis of comorbid conditions

The NLRP3 inflammasome has emerged as a central inflammatory hub that may link myocardial infarction and depression through interconnected mechanisms forming a self-amplifying network between the heart and the brain. The following subsections systematically examine these pathways, from upstream triggers to downstream effectors and systemic modulators. Before detailing individual mechanisms, it is important to note that the vast majority of evidence reviewed below derives from single-disease models either MI or depression. Where direct evidence in comorbidity-specific systems exists, we explicitly highlight it, otherwise, the pathways are presented as mechanistically plausible links that await causal validation in integrated heart-brain models.

### 5.1 Oxidative stress and mitochondrial dysfunction

Oxidative stress and mitochondrial dysfunction are considered core upstream events in the activation of the NLRP3 inflammasome in the comorbidity of MI and depression (Figure 1). During the ischemia-reperfusion process following MI, damaged cardiomyocytes release large amounts of mitochondrial reactive oxygen species (mtROS) and mitochondrial DNA (mtDNA), which can act as damage-associated molecular patterns (DAMPs) that may be recognized by the NLRP3 inflammasome and trigger its assembly and activation (54). Concurrently, chronic stress also induces mitochondrial dysfunction in the central nervous system, promoting excessive mtROS production and activating the NLRP3 inflammasome in microglia, thereby potentially mediating neuroinflammatory responses (24, 54, 55).

**Figure 1:**
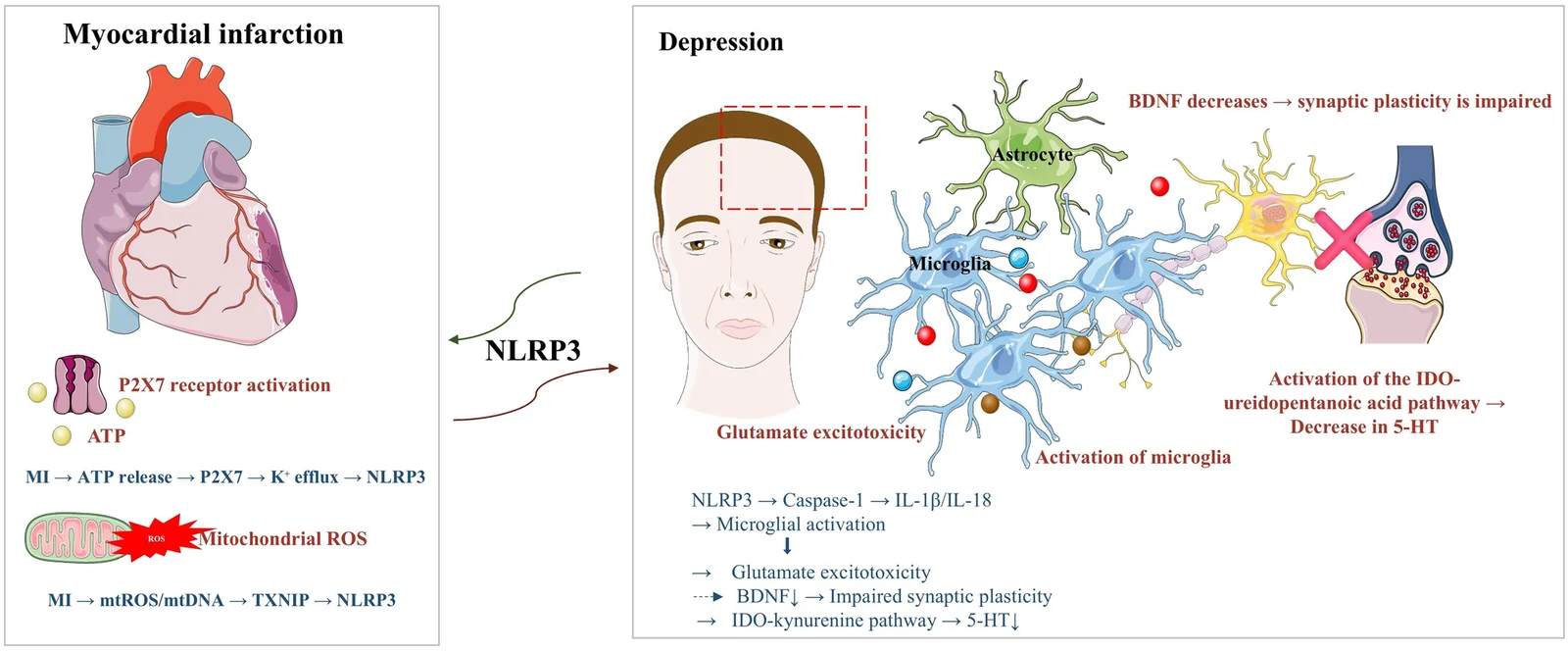
Mechanisms of myocardial infarction and depression comorbidity via the NLRP3 inflammasome. After myocardial infarction (MI), damaged myocardial cells release a large amount of injury-related molecular patterns, among which extracellular ATP and mitochondrial reactive oxygen species (mitochondrial ROS) are the key upstream signals that activate the NLRP3 inflammasome. ATP activates the P2X7 receptor, inducing the production of mitochondrial ROS, and jointly promote the assembly and activation of the NLRP3 inflammasome. The activated NLRP3 inflammasome further activates microglia, initiating central nervous inflammation. Activated microglia mediate depressive behaviors through two main pathways: first, activating the indoleamine 2,3-dioxygenase (IDO)-tryptamine pathway, which leads to the diversion of tryptophan metabolism and reduces the synthesis of 5-hydroxytryptamine (5-HT). Second, inducing glutamate excitotoxicity, causing excessive excitation and damage to neurons. At the same time, the activation of the NLRP3 inflammasome also leads to dysfunction of astrocytes, reducing the expression of brain-derived neurotrophic factor (BDNF), and thereby damaging synaptic plasticity. The combined effects of 5-HT reduction, glutamate excitotoxicity, and BDNF downregulation ultimately lead to the occurrence of depressive behaviors. This figure systematically demonstrates the complete signal cascade reaction from myocardial infarction to NLRP3 inflammasome activation, to neurotransmitter metabolic disorders and synaptic damage.

At the molecular level, mtROS bind to NLRP3 via thioredoxin-interacting protein (TXNIP), while mtDNA engages TLR9 to provide a priming signal. When mitophagy is impaired, damaged mitochondria accumulate and continuously release mtROS and mtDNA, leading to persistent NLRP3 inflammasome activation (56, 57). Thus, mitochondrial dysfunction represents a convergent upstream trigger for NLRP3 activation in both the injured heart and the stressed brain, establishing a mechanistic link between cardiac ischemia and neuroinflammation.

Pharmacological activation of SIRT1 has been shown to suppress NLRP3 inflammasome activation and improve both cardiac and depressive outcomes in comorbid models. In a rat model of comorbid MI and depression, SIRT1 upregulation by dapagliflozin reduced pyroptosis, improved cardiac function, and ameliorated depressive-like behaviors, with *in vitro* experiments confirming that this beneficial effect is dependent on the SIRT1/NLRP3 signaling pathway (58). Enhancement of mitophagy represents another mechanism by which NLRP3 inflammasome activation is inhibited. Treatment with paeoniflorin promoted mitochondrial autophagy, restored mitochondrial membrane potential, and reduced NLRP3, ASC, caspase-1, and IL-1β expression in CUMS-induced mice, accompanied by alleviation of depressive-like behaviors and hippocampal neuronal damage (59, 60). Similar inhibition of NLRP3 inflammasome activation through paeoniflorin has been observed in models of acute MI and diabetic cardiomyopathy, demonstrating cardioprotective effects (61, 62). Collectively, these observations suggest that enhancing mitochondrial quality control mechanisms represents a promising strategy for breaking the NLRP3-mediated vicious cycle between cardiac injury and depressive neuroinflammation.

### 5.2 ATP-P2X7 signaling axis and Ca^2^⁺ signaling pathway

Extracellular ATP, as a damage-associated molecular pattern, is released under conditions of tissue injury, inflammation, and stress, and can trigger NLRP3 inflammasome assembly and activation through activation of the purinergic P2X7 receptor [63–70]. The P2X7 receptor is an ATP-gated cation channel widely expressed in immune cells and the cardiovascular system (65, 66). Unlike other P2X subtypes, it has a low affinity for ATP, requiring high pathological concentrations for effective activation, making it a specific sensor of tissue injury. The channel also exhibits minimal desensitization and can transition into a large pore upon prolonged stimulation (67).

In the context of MI, the ATP-P2X7 signaling axis exerts complex bidirectional regulatory effects. However, the precise bidirectional relationship between ATP release and P2X7 activation in the ischemic heart remains to be fully established (71–74). One study using a C57BL/6J mouse MI model induced by left anterior descending coronary artery ligation demonstrated that P2X7 receptor and NLRP3 gene silencing or pharmacological inhibition prevented NLRP3 inflammasome formation, reduced infarct size, and attenuated cardiac enlargement (73, 74). In a diabetic myocardial ischemia-reperfusion injury model, investigators induced diabetes in C57BL/6J mice using a high-fat diet combined with streptozotocin, followed by myocardial ischemia-reperfusion surgery (73). Results showed that P2X7 expression was upregulated and inflammatory cell infiltration was aggravated in the myocardial tissue of diabetic mice. Treatment with the P2X7 antagonists brilliant blue G or A438079 significantly reduced infarct size, improved cardiac function, and inhibited apoptosis (73). These observations suggest a role for P2X7 signaling in MI, but the bidirectional regulatory dynamics require further investigation.

The P2X7 receptor has been proposed as a potential interface molecule connecting the nervous, immune, and cardiovascular systems. However, direct evidence from the cardiovascular system supporting this role remains limited, and further investigation is needed to establish its significance in heart-brain comorbidity. In depression, chronic stress induces ATP release, activating microglial P2X7 and triggering NLRP3 inflammasome assembly and IL-1β/IL-18 secretion (75). Constitutive P2X7 knockout mice exhibit enhanced stress resilience (76), while astrocyte-specific P2X7 knockdown in hippocampal CA1 mediates acute stress-induced depressive-like responses (77). In heart-brain comorbidity, the ATP-P2X7-Ca^2^⁺ axis has been hypothesized to link myocardial injury and neuroinflammation, although direct evidence for this pathway in comorbid systems remains limited. Post-MI ATP release may influence myocardial inflammation through P2X7, and circulating ATP may affect central inflammation via indirect mechanisms, though direct evidence for ATP crossing the BBB remains to be determined (64, 72).

Several unresolved issues temper translational optimism. P2X7 exhibits context-dependent duality, promoting repair in some settings while exacerbating injury in others, and this pleiotropy remains uninvestigated in comorbidity models. The relative contributions of P2X7 on different cell types have not been dissected using cell-specific knockouts. ATP concentrations required for P2X7 activation exceed typical extracellular levels, raising questions about direct vs. indirect mechanisms. P2X7 antagonists with varying selectivity profiles have not been compared head-to-head in comorbidity models, and no clinical biomarker study has linked P2X7 to post-MI depression risk or treatment response (64, 72, 73, 76, 77).

### 5.3 Peripheral and central inflammation

At the cellular level, peripheral and central inflammation crosstalk is a key pathological feature of MI-depression comorbidity. MI activates the peripheral innate immune system, and inflammatory signals are subsequently transmitted to the central nervous system (CNS) through multiple pathways, inducing neuroinflammation and depressive-like behaviors (78–80). Following MI, damaged cardiomyocytes release DAMPs including HMGB1, S100 proteins, extracellular ATP, and mitochondrial DNA, which activate resident immune cells via TLR2, TLR4, and P2X7 receptors, triggering NLRP3 inflammasome assembly and IL-1β/IL-18 secretion (78–80). Inflammatory signals reach the CNS through three routes: cytokine transport across the BBB, peripheral immune cell infiltration, and vagal afferent signaling via the nucleus tractus solitarius (81, 82).

Peripheral-to-central inflammatory transmission following MI is mediated in part by microglial NLRP3 activation. In post-MI depression models, elevated pro-inflammatory cytokines in plasma and hippocampus are accompanied by increased microglial activation and upregulated NLRP3, ASC, and caspase-1 in the hippocampus, correlating with impaired cardiac function and depressive-like behaviors. Inhibition of microglial NLRP3 inflammasome activation through minocycline intervention reversed these pathological changes, confirming that microglial NLRP3 activation serves as a key link between cardiac injury and depressive behavior (83). Additionally, cardiac-derived extracellular vesicles have been identified as mediators of heart-brain communication, as MI-derived extracellular vesicles induced hippocampal NLRP3 activation and depressive-like behaviors in naïve mice through the TLR4/NLRP3 axis, an effect blocked by the TLR4 inhibitor TAK-242 (84, 85).

Mitochondrial dysfunction may mediate peripheral-central inflammatory crosstalk (86). Following MI, mtDNA and mtROS from damaged cardiomyocytes activate peripheral immune cells and can enter the brain, where microglial phagocytosis triggers endogenous NLRP3 inflammasome activation (87). Post-MI systemic redox imbalance may also compromise BBB integrity, facilitating inflammatory cell migration into the CNS. Peripheral and central inflammation form a bidirectional positive feedback loop. Peripheral signals activate microglia via direct CNS entry or vagal afferent pathways, and activated microglia release IL-1β, IL-18, and TNF-α, which act on peripheral tissues through HPA axis stimulation and autonomic nervous system modulation, increasing sympathetic tone and reducing heart rate variability. Thus, the cellular mechanisms described here operate in coordination with molecular signals and neural pathways to form an integrated network linking cardiac injury to central neuroinflammation. While vagal afferent signaling provides a route for peripheral inflammatory signals to reach the CNS, the neural modulation of NLRP3 inflammasome activity through sympathetic and parasympathetic efferent pathways is discussed in Section 5.4.

### 5.4 Sympathetic nervous system overactivation and vagus nerve-mediated inflammatory reflex pathway

While Section 5.3 focused on how peripheral inflammatory signals reach the CNS, this section addresses the neural efferent pathways that modulate NLRP3 inflammasome activity and inflammation. The autonomic nervous system regulates cardiovascular function and immune responses, and its dysregulation is a key mechanism linking MI and depression. Sympathetic overactivation and parasympathetic inhibition, particularly the vagus-mediated cholinergic anti-inflammatory pathway, constitute a neural circuit for peripheral-central inflammatory interaction in heart-brain comorbidity.

Following MI, decreased cardiac output and baroreceptor unloading trigger sympathetic overactivation, which elevates myocardial oxygen consumption and promotes ventricular remodeling (88). Norepinephrine acts on immune cells via β-adrenergic receptors and may enhance NLRP3 inflammasome activation. Sympathetic overactivation enhances NLRP3 inflammasome activation through β-adrenergic receptor signaling. In obese rats, increased cardiac sympathetic activity correlates with upregulated NLRP3 and IL-1β expression and increased CD68⁺NLRP3⁺ macrophage infiltration (88). *In vitro*, norepinephrine promotes THP-1 macrophage polarization toward a pro-inflammatory phenotype and NLRP3 activation, an effect dependent on protein kinase A activation and subsequent β-arrestin2 inhibition (89, 90). Sympathetic overactivation may also impair BBB integrity, facilitating peripheral inflammatory factor entry into the CNS. In depression, chronic stress induces HPA axis dysfunction and increased sympathetic tone, elevating norepinephrine levels that activate NLRP3 inflammasomes in microglia and astrocytes, driving neuroinflammation and depressive-like behaviors (91, 92). Sympathetic signals also influence peripheral immune cells through secondary lymphoid organs such as the spleen (93).

The vagus-mediated cholinergic anti-inflammatory pathway opposes sympathetic effects. Acetylcholine binds α7 nicotinic acetylcholine receptors on immune cells, inhibiting NF-κB nuclear translocation and JAK-STAT signaling, thereby downregulating NLRP3-related proteins and suppressing pro-inflammatory cytokine production (94, 95). Reduced vagal tone post-MI is associated with worsening cardiac function and elevated inflammation. Clinical studies have shown that reduced heart rate variability in MI patients predicts post-MI depression, suggesting vagal activity partially protects against psychological distress (96). Magnetic vagus nerve stimulation in a rat I/R injury model reduced infarct size, decreased ventricular fibrillation, and inhibited inflammatory cytokine release. Mechanistically, it inhibited NLRP3-mediated pyroptosis through the M_2_ acetylcholine receptor/OGDHL/ROS axis, reducing NLRP3 expression, cleaved caspase-1 and GSDMD levels, and IL-1β/IL-18 release, while improving mitochondrial function (97). In comorbidity, sympathetic-vagal imbalance forms a positive feedback loop: sympathetic overactivation exacerbates NLRP3 activation and inflammatory release, while inflammatory factors further stimulate sympathetic nerves and inhibit vagal function. This autonomic-immune-inflammatory dysregulation exacerbates myocardial injury, cardiac remodeling, and neuroinflammation. Restoring autonomic balance, enhancing vagal tone, or blocking sympathetic signaling may represent therapeutic strategies. Vagus nerve stimulation, β-blockers, and alpha-7 nicotinic acetylcholine receptor (α7nAChR) agonists have shown preclinical efficacy, but clinical validation in comorbid patients is needed.

### 5.5 Gut microbiota-gut-brain axis pathway

The gut microbiota-gut-brain axis has been proposed as a potential pathway linking MI and depression, although it should be emphasized that direct evidence in the context of heart-brain comorbidity remains limited. The majority of the findings discussed below derive from preclinical models and represent a promising but preliminary area of investigation that requires further validation in comorbid systems. Gut microbiota dysbiosis participates in cardiovascular diseases and depression, with the NLRP3 inflammasome serving as a molecular node in this bidirectional network (Figure 2). Gut microbiota-derived signals are sufficient to transmit depressive-like phenotypes, as demonstrated by fecal microbiota transplantation from depressive-like MI rats into pseudo-sterile recipients, which induced depressive-like behaviors and altered gut microbiota composition (98). Similarly, gut microbiota from depressed patients transmitted depressive-like behaviors to recipient animals, confirming the causal role of gut microbial signals in depression pathogenesis (99). Patients with comorbid MI and depression exhibit reduced microbial diversity, decreased beneficial bacteria such as Faecalibacterium and Roseburia, and increased pro-inflammatory Enterobacteriaceae (100, 101). This dysbiosis impairs intestinal barrier function, allowing lipopolysaccharide translocation into the bloodstream and inducing systemic low-grade inflammation (100, 101).

**Figure 2:**
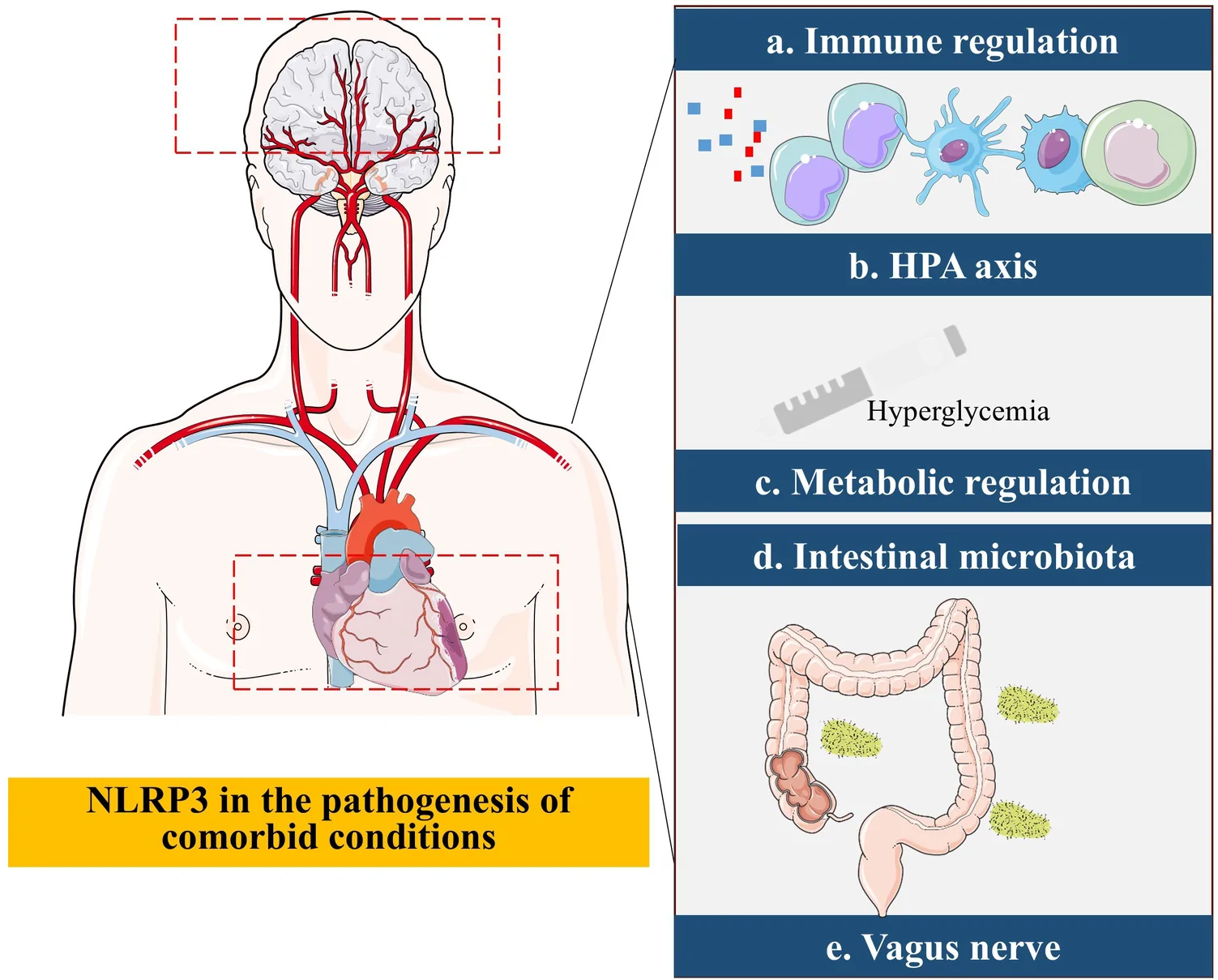
Multidimensional regulatory network of the NLRP3 inflammasome in the pathogenesis of MI-depression comorbidity. The NLRP3 inflammasome plays a central regulatory role in the pathogenesis of myocardial infarction (MI) and depression comorbidity through five interconnected dimensions. **(a)** Immune regulation: Activation of the NLRP3 inflammasome promotes the maturation and release of IL-1β and IL-18, induces GSDMD-mediated pyroptosis, and activates microglia through peripheral-central inflammatory crosstalk, driving neuroinflammatory responses. **(b)** HPA axis: Inflammatory cytokines stimulate the hypothalamic-pituitary-adrenal (HPA) axis, leading to abnormal cortisol secretion, which in turn can activate the NLRP3 inflammasome, forming a positive feedback loop. **(c)** Metabolic regulation: Metabolic disturbances such as hyperglycemia activate the NLRP3 inflammasome through mechanisms including mitochondrial ROS production and MARK4-dependent microtubule dynamics; conversely, NLRP3 activation exacerbates insulin resistance and lipid metabolism abnormalities, creating a metabolic-inflammatory vicious cycle. **(d)** Intestinal microbiota: Post-MI intestinal ischemia leads to gut dysbiosis; lipopolysaccharide (LPS) enters the circulation and activates the NLRP3 inflammasome via TLR4, while reduced short-chain fatty acids (SCFAs) weaken their inhibitory effect on NLRP3. **(e)** Vagus nerve: The vagus nerve-mediated cholinergic anti-inflammatory pathway inhibits NF-κB nuclear translocation and NLRP3 inflammasome activation through α7 nicotinic acetylcholine receptors; post-MI reduction in vagal tone removes this inhibition, exacerbating inflammatory responses. These five dimensions do not act independently but are interwoven and synergistically amplified, collectively constituting the NLRP3 inflammasome-mediated network of heart-brain comorbidity. Targeting any of these dimensions or directly inhibiting the NLRP3 inflammasome holds potential to interrupt the bidirectional vicious cycle between MI and depression. Correspondence between the five dimensions **(a–e)** in this figure and the main text sections: **(a)** Immune regulation corresponds to Section 5.3 (peripheral and central inflammation); **(b)** HPA axis corresponds to Section 5.4 (sympathetic and vagus nerve pathways); **(c)** Metabolic regulation corresponds to Section 5.8 (metabolic dysregulation); **(d)** Intestinal microbiota corresponds to Section 5.5 (gut microbiota-gut-brain axis); **(e)** Vagus nerve corresponds to Section 5.4 (sympathetic and vagus nerve pathways).

Gut microbial metabolites mediate heart-brain interactions. Short-chain fatty acids activate G protein-coupled receptors 41/43, inhibit histone deacetylases, suppress NLRP3 inflammasome activation, and alleviate neuroinflammatory responses (102). Sodium butyrate improves neurological outcomes in cardiac arrest mice and inhibits M1 microglial polarization (103). *In vitro*, a short-chain fatty acid mixture reduces IL-1β and TNF-α secretion and inhibits ROS production (104). Post-MI gut dysbiosis arises from reduced cardiac output and mesenteric blood flow causing intestinal ischemia and mucosal injury (105), with ischemia-reperfusion-generated ROS disrupting epithelial tight junctions and promoting pathogen overgrowth. Stress-induced sympathetic excitation and cortisol release further affect intestinal motility and microbiota composition (106). These changes remodel gut microbiota within hours after MI, exacerbating post-MI inflammation and depression (107, 108). Interventions targeting the gut-brain axis have shown promise. Probiotics containing Lactobacillus reuteri R0052 and Bifidobacterium longum R0175 reduced immobility time and improved social interaction in MI rats (109). High n-3 polyunsaturated fatty acid diets or probiotics immediately after reperfusion alleviated post-MI depressive-like behaviors (110). Postbiotics and TMAO pathway inhibitors have also demonstrated preclinical potential (111). Combining probiotics with NLRP3 inhibitors may yield synergistic effects by restoring microbial balance while blocking inflammatory cascades.

In summary, the gut microbiota-gut-brain axis has been proposed to connect MI and depression through NLRP3 inflammasome-mediated immuno-inflammatory pathways, constituting a potential mechanism of heart-brain interaction. Fecal microbiota transplantation experiments have suggested that post-MI gut microbiota dysbiosis may contribute to depressive-like behaviors, and metabolites such as short-chain fatty acids and lipopolysaccharide have been reported to mediate heart-brain signal transmission by regulating microglial polarization and NLRP3 activation.

### 5.6 Neurotransmitter and neurotrophic metabolic dysregulation

Importantly, abnormalities in neurotransmitter and neurotrophic factor metabolism are observed across diverse depression models and are not specific to post-MI depression. The following discussion prioritizes evidence from post-MI depression models and MI-chronic stress comorbidity models, and explicitly distinguishes these from evidence derived from general depression models. Depression after MI may result from both biological and psychosocial factors, and the mechanisms discussed below should be interpreted accordingly.

NLRP3 inflammasome activation regulates serotonergic and glutamatergic systems and BDNF signaling in post-MI depression (Table 1). Following MI, systemic inflammation activates the IDO-kynurenine pathway, diverting tryptophan from serotonin synthesis and generating neurotoxic metabolites such as quinolinic acid, which produce excitotoxicity via NMDA receptor activation (112). In comorbid rat models, hippocampal and prefrontal IDO activity and kynurenine/tryptophan ratio were elevated with decreased serotonin levels, and the IDO inhibitor 1-methyltryptophan partially reversed depressive-like behaviors (112, 113).

**Table 1:** Core mechanisms of NLRP3 inflammasome-mediated bidirectional induction between MI and depression.

Mechanism category	Post-MI changes	Depression-related changes	Shared pathways/key molecules	Refs.
Oxidative stress and mitochondrial dysfunction	Ischemia-reperfusion releases mtROS (peak at 2–6 h post-reperfusion) and mtDNA from cardiomyocytes; activates NLRP3 in macrophages and cardiomyocytes	Chronic stress induces CNS mitochondrial dysfunction (observed in hippocampus within 7–14 days of stress); mtROS activates microglial NLRP3	mtROS-mtDNA-TXNIP-NLRP3 axis	(11, 24, 54–56)
ATP-P2X7-Ca^2^⁺ signaling axis	Necrotic myocardium releases ATP (sustained elevation in infarct zone); activates P2X7 on macrophages and cardiomyocytes	Chronic stress elevates extracellular ATP in prefrontal cortex and hippocampus (detected after 21 days of CUMS); activates microglial P2X7 receptors	ATP-P2X7-K⁺ efflux-NLRP3 axis	(63, 64, 67, 69, 71, 75, 76)
Peripheral and central inflammation crosstalk	Post-MI DAMPs enter brain via circulation; TLR4 peaks at 1 h (protein) and 4 h (mRNA) post-reperfusion; IL-1β rises within 1 h and peaks at 12 h; activates microglia in hippocampus and PFC	Central inflammatory cytokines affect cardiovascular function via HPA axis and autonomic nerves (observed after 14–28 days of stress)	TLR4/NLRP3-mediated peripheral-central crosstalk	(78–83, 85)
Sympathetic and vagus nerve pathways	Post-MI sympathetic overactivation (increased NE within 24 h); β-AR enhances NLRP3 in cardiac macrophages	Chronic stress increases sympathetic tone (elevated NE in brain at day 14–28); activates microglial NLRP3	β-AR/cAMP/PKA signaling pathway	(88–92)
Gut microbiota-gut-brain axis	Post-MI intestinal ischemia causes dysbiosis within 6–12 h; Gram-negative bacterial translocation elevates plasma LPS (detectable at 24 h)	Gut dysbiosis in depression (observed in CUMS models after 21–28 days); LPS induces peripheral and central inflammation	LPS-TLR4-NLRP3 axis	(98–101)
Neurotransmitter and neurotrophic factors	Peripheral/central inflammation activates IDO within 6–24 h; reduces 5-HT in hippocampus and PFC	IDO activation depletes tryptophan (detectable at day 7–21); quinolinic acid induces excitotoxicity	IDO-kynurenine-quinolinic acid pathway	(112, 114, 115)
Epigenetic remodeling	Histone modifications (acetylation at H3K9) upregulate NLRP3 expression in macrophages within 24 h	Hypermethylation of BDNF promoter (observed in hippocampus at day 14–28) reduces neurotrophic support	NLRP3 promoter methylation, histone acetylation	(120, 121)

MI, myocardial infarction; mtROS, mitochondrial reactive oxygen species; mtDNA, mitochondrial DNA; TXNIP, thioredoxin-interacting protein; CNS, central nervous system; HPA, hypothalamic-pituitary-adrenal; DAMP, damage-associated molecular pattern; TLR4, toll-like receptor 4; β-AR, β-adrenergic receptor; LPS, lipopolysaccharide; IDO, indoleamine-2,3-dioxygenase; 5-HT, serotonin; BDNF, brain-derived neurotrophic factor.

Glutamatergic dysfunction also mediates NLRP3 involvement. Activated microglia and astrocytes release excess glutamate while downregulating GLT-1 and GLAST transporters, causing synaptic accumulation. Excessive glutamate overactivates NMDA receptors, triggering Ca^2^⁺ influx and mitochondrial dysfunction, which further activates NLRP3 (114). Quinolinic acid acts as an endogenous NMDA agonist, and the antagonist MK-801 attenuated neuroinflammation and depressive-like behaviors in I/R models (114, 115). BDNF signaling inhibition is a core mechanism of NLRP3-mediated neuroplasticity damage. Pro-inflammatory cytokines inhibit Bdnf transcription via NF-κB, and caspase-1 cleaves the BDNF precursor (115). Serum BDNF is reduced in post-MI depression and correlates negatively with severity. In comorbid rats, hippocampal BDNF was downregulated, and the TrkB agonist 7,8-dihydroxyflavone ameliorated depressive-like behaviors (115, 116). BDNF and NLRP3 exhibit bidirectional regulation: cytokines suppress BDNF, while BDNF inhibits microglial NLRP3 activation via PI3 K/Akt and MAPK/ERK pathways (116). BDNF preconditioning in I/R models reduced infarct size, lowered NLRP3 activation, and improved cardiac function and depressive outcomes (117, 118).

Noradrenergic overactivation enhances NLRP3 through β-adrenergic receptors, dopaminergic dysfunction correlates with anhedonia, and reduced GABAergic inhibition contributes to excitation/inbalance (119). BDNF promoter methylation correlates with depression severity, and polymorphisms rs908867 and rs925946 affect cognition via methylation (120, 121). Several limitations remain. The IDO pathway has been studied primarily with 1-methyltryptophan despite off-target effects. Glutamatergic studies relied on MK-801, which is clinically unsuitable. BDNF measurements are inconsistent across assays, and peripheral-central relationships are unclear. The BDNF-NLRP3 regulatory loop requires mechanistic elucidation, and genetic variation in BDNF and related genes has been largely unexplored in comorbidity studies (115, 116, 120, 121).

In summary, the NLRP3 inflammasome participates in the pathogenesis of post-MI depression by regulating multiple neurotransmitter systems, including the serotonergic and glutamatergic systems, and BDNF neurotrophic signaling, mediating synaptic plasticity impairment and neuronal dysfunction. Targeting the IDO-kynurenine pathway, NMDA receptors, and the BDNF-TrkB signaling pathway may represent novel therapeutic strategies for post-MI depression. However, the fine regulatory networks between these neurotransmitters and neurotrophic factors and the NLRP3 inflammasome, as well as their specific roles in heart-brain interactions, require further elucidation. Direct evidence linking MI itself to these neurotransmitter changes, independent of chronic stress, remains limited and warrants further investigation.

### 5.7 VEGF-mediated alterations in vascular permeability and abnormalities in angiogenic signaling

Vascular endothelial growth factor (VEGF) serves as a critical molecular node connecting the comorbidity of MI and depression. Following MI, VEGF regulates BBB permeability and neurovascular remodeling, thereby participating in the development of depressive behaviors. The VEGF family includes VEGF-A, VEGF-B, VEGF-C, VEGF-D, VEGF-E, and placental growth factor, among which VEGF-A is the most extensively studied prototypic member (122). VEGF exerts its biological effects by binding to its receptors VEGFR1, VEGFR2, and VEGFR3, with VEGFR2 serving as the primary receptor mediating vascular permeability and neuroprotective effects (123).

VEGF signaling exerts bidirectional regulatory effects following MI. Early post-MI VEGF promotes compensatory angiogenesis and collateral circulation establishment, while excessive VEGF expression impairs BBB integrity. NLRP3 inflammasome activation-induced vascular damage alters BBB permeability, causes endothelial dysfunction, and facilitates peripheral inflammatory factor entry into the central nervous system (124). In atherosclerosis, the NLRP3 inflammasome serves as a key mediator of vascular inflammation (122, 125).

The role of VEGF in MI depression comorbidity is the least well characterized among the seven pathways reviewed, and the available evidence is predominantly inferential rather than direct. No study has yet systematically measured VEGF expression, receptor phosphorylation, or downstream signaling in both cardiac and cerebral tissues across the acute and chronic phases of MI depression comorbidity in the same experimental paradigm. The temporal relationship between VEGF dysregulation and NLRP3 activation, whether VEGF is an upstream trigger, a downstream consequence, or a parallel independent pathway, therefore remains entirely uncharacterized.

### 5.8 Metabolic dysregulation

Metabolic dysregulation represents one of the core pathological foundations connecting myocardial infarction (MI) and depression comorbidity. The NLRP3 inflammasome plays a critical role in metabolic diseases such as obesity, insulin resistance, type 2 diabetes mellitus, and atherosclerosis by sensing metabolic stress signals (126). These metabolic disorders serve as both independent risk factors for MI and contributors to the pathogenesis of depression. Post-MI metabolic abnormalities can further exacerbate NLRP3 inflammasome activation, forming a vicious cycle of heart-brain mutual reinforcement (Table 2).

**Table 2:** Key NLRP3 inflammasome-related signaling pathways and molecular targets in MI-depression comorbidity.

Signaling pathway	Role in comorbidity	Key upstream signals	Key downstream effects	Targeted drugs	Refs.
TLR4/NF-κB	DAMPs/PAMPs activate TLR4, priming NLRP3 and pro-IL-1β transcription	DAMPs (HMGB-1, S100 proteins), LPS (1 μg/mL); TLR4 peaks at 1 h (protein) and 4 h (mRNA) post-reperfusion; LPS induces NF-κB nuclear translocation within 10–30 min in macrophages/microglia	NF-κB nuclear translocation (maximal by 30 min); upregulates NLRP3 mRNA (2–5 fold increase at 6–24 h) in macrophages and microglia	TAK-242 (TLR4 inhibitor), curcumin, metformin	(24, 78–80)
ATP-P2X7	Extracellular ATP activates P2X7, triggering K⁺ efflux and NLRP3 assembly	Extracellular ATP (sustained elevation in infarct zone post-MI); Intracellular K⁺ efflux is required for NLRP3 activation	ASC oligomerization (within 5–15 min of ATP stimulation); caspase-1 activation (cleavage at 30 min–2 h) in macrophages/microglia	Brilliant Blue G, A438079, JNJ-47965567	(63, 64, 67, 69, 73)
Mitochondrial ROS-TXNIP	ROS oxidizes TXNIP, relieving its inhibition of NLRP3	mtROS (peak at 2–6 h post-reperfusion; >2-fold increase over baseline); mtDNA released from damaged mitochondria	Conformational change of NLRP3; inflammasome assembly (1–4 h post-stimulation) in cardiomyocytes, macrophages, and microglia	MitoTEMPO, MCC950, INF4E	(24, 54–56, 87)
NLRP3 Inflammasome	Central inflammatory amplifier in the comorbidity process	Priming signal (TLR4/NF-κB, 1–6 h) + activation signal (ATP/P2X7 or ROS/TXNIP, 5–30 min)	IL-1β rises within 1 h post-MI and peaks at 12 h (570.58 ± 31.19 pg/mL); IL-18 maturation; pyroptosis via GSDMD-NT pore formation (30–60 min; cell death at 2–4 h)	MCC950, INF4E/INF200, OLT1177, BGE-102, MRT-8102, Selnoflast, VX-765	(15, 17, 24, 29, 80, 130)
PI3 K/Akt/mTOR	Regulates autophagy and NLRP3 activity, affecting cell survival	Growth factors (IGF-1), insulin; pathway activation within 5–30 min in cardiomyocytes and microglia	Altered autophagic flux (LC3-II/I ratio changes at 1–4 h); NLRP3 degradation via autophagy (lysosomal co-localization at 2–6 h)	Rapamycin, LY294002, metformin	(52, 56, 57, 59, 60, 137)
MAPK (p38/JNK/ERK)	p38/JNK promote inflammation and apoptosis; ERK has dual effects	Stress signals (ROS), pro-inflammatory cytokines (TNF-α); activation within 5–30 min in macrophages/microglia	AP-1 activation (peaks at 1–2 h); pro-inflammatory cytokine production (IL-6, TNF-α at 2–6 h) in macrophages/microglia/cardiomyocytes	SB203580 (p38 inhibitor), SP600125 (JNK inhibitor)	(116, 128)
JAK/STAT	Mediates cytokine signaling, regulates inflammation and neuroplasticity	IL-6, IFN-γ; STAT3 phosphorylation within 15–60 min in microglia and macrophages	STAT3 phosphorylation; pro- or anti-inflammatory gene transcription (1–4 h); regulates SOCS3 expression and NLRP3 activity	AG490 (JAK2 inhibitor)	(94, 112, 128)
Autophagy	Clears damaged mitochondria, limits NLRP3 activation; impaired autophagy promotes inflammation	mTOR inhibition (rapamycin 100 nM, 90 min pretreatment); AMPK activation; Beclin1 upregulation (1–4 h)	Altered LC3-II/p62 ratio (LC3-II increase ≥2-fold; p62 decrease ≥50% at 2–6 h); inflammasome degradation via autophagolysosomes (2–6 h)	Rapamycin, 3-methyladenine	(52, 56, 59)

DAMP, damage-associated molecular pattern; PAMP, pathogen-associated molecular pattern; TLR, toll-like receptor; MyD88, myeloid differentiation primary response 88; IKK, IκB kinase; NF-κB, nuclear factor-κB; ROS, reactive oxygen species; TXNIP, thioredoxin-interacting protein; ASC, apoptosis-associated speck-like protein containing a CARD domain; IL, interleukin; GSDMD, gasdermin D; PI3K, phosphoinositide 3-kinase; Akt, protein kinase B; mTOR, mechanistic target of rapamycin; MAPK, mitogen-activated protein kinase; JNK, c-Jun N-terminal kinase; ERK, extracellular signal-regulated kinase; AP-1, activator protein-1; JAK, Janus kinase; STAT, signal transducer and activator of transcription; IFN, interferon.

Metabolic stress signals serve as potent NLRP3 activators in both peripheral macrophages and central microglia. Obesity-induced adipose tissue inflammation generates cholesterol crystals and lipid derivatives that activate the NLRP3 inflammasome in macrophages (24, 126). These metabolic abnormalities accelerate atherosclerosis and affect CNS function through systemic inflammation (24, 126). Cholesterol crystals and fatty acid derivatives trigger TLR4 signaling, inducing Ca^2^⁺ influx, ROS production, and release of TNF-α, IL-6, and MCP-1. These mediators enter the circulation, cross the BBB, activate microglial NLRP3, and drive neuroinflammatory responses and depressive-like behaviors. Clinical studies have shown that MI patients with metabolic syndrome have a higher incidence of depression, suggesting metabolic dysregulation contributes to post-MI depression. Under hyperglycemic conditions, NLRP3 is activated through MARK4-dependent microtubule dynamics and increased mitochondrial ROS production (127).

Metabolic reprogramming regulates NLRP3 activation through multiple mechanisms. Enhanced glycolysis promotes lactate-EIF2AK2 signaling and transcriptional induction of glycolytic genes via the PKM2 and HK1-mTORC1 axis. Succinate accumulation under hypoxic stress stabilizes HIF-1α to promote NLRP3 activation, while itaconate acts as an endogenous inhibitor by covalently modifying NLRP3 and disrupting NEK7 binding (128, 129). Post-MI myocardial ischemia leads to local and systemic metabolic reprogramming, with enhanced glycolysis and lactate accumulation affecting both cardiac repair and CNS metabolic environment, exacerbating depressive symptoms.

Post-MI insulin resistance is common, with reduced glucose uptake and elevated free fatty acids further activating NLRP3. MI patients with insulin resistance have elevated serum IL-1β and IL-18 levels, which correlate positively with depression scores. High-fat diet-induced insulin-resistant mice exhibit more severe depressive-like behaviors and hippocampal NLRP3 activation following MI. A bidirectional relationship exists between MI and metabolic dysregulation. Post-MI cardiac decline reduces physical activity and energy expenditure, promoting obesity and insulin resistance. Conversely, metabolic dysregulation accelerates atherosclerosis via NLRP3-mediated inflammation and exacerbates depressive symptoms through neuroinflammation. This vicious cycle promotes comorbidity progression.

### 5.9 Integrated mechanistic framework: a summary

The seven pathways described above form an interconnected and self-amplifying network centered on the NLRP3 inflammasome, operating through three hierarchical levels. At the primary level, initiating triggers including mitochondrial dysfunction and ATP release converge on NLRP3 inflammasome assembly. At the secondary level, NLRP3 activation amplifies inflammation through IL-1β/IL-18 secretion, IDO-kynurenine pathway activation, and BDNF suppression, establishing bidirectional heart-brain crosstalk and impairing synaptic plasticity. At the tertiary level, systemic modulators such as autonomic imbalance, gut dysbiosis, and VEGF-mediated vascular changes sustain NLRP3 activation over time, contributing to the chronic and recurrent nature of post-MI depression and cardiac remodeling.

Critical questions remain regarding the temporal sequence and relative contribution of each pathway. Longitudinal studies with repeated measurements across multiple time points and sequential pathway blockade are needed to address these gaps. Therapeutically, if pathways are interdependent, single-target intervention may yield limited benefit, whereas identification of a dominant pathway could enable more effective targeting. This integrated view reframes the comorbidity as a self-sustaining inflammatory loop in which cardiac injury triggers central inflammation, which in turn exacerbates cardiac dysfunction, suggesting that optimal therapeutic strategies may need to be stage-dependent (Figure 3).

**Figure 3:**
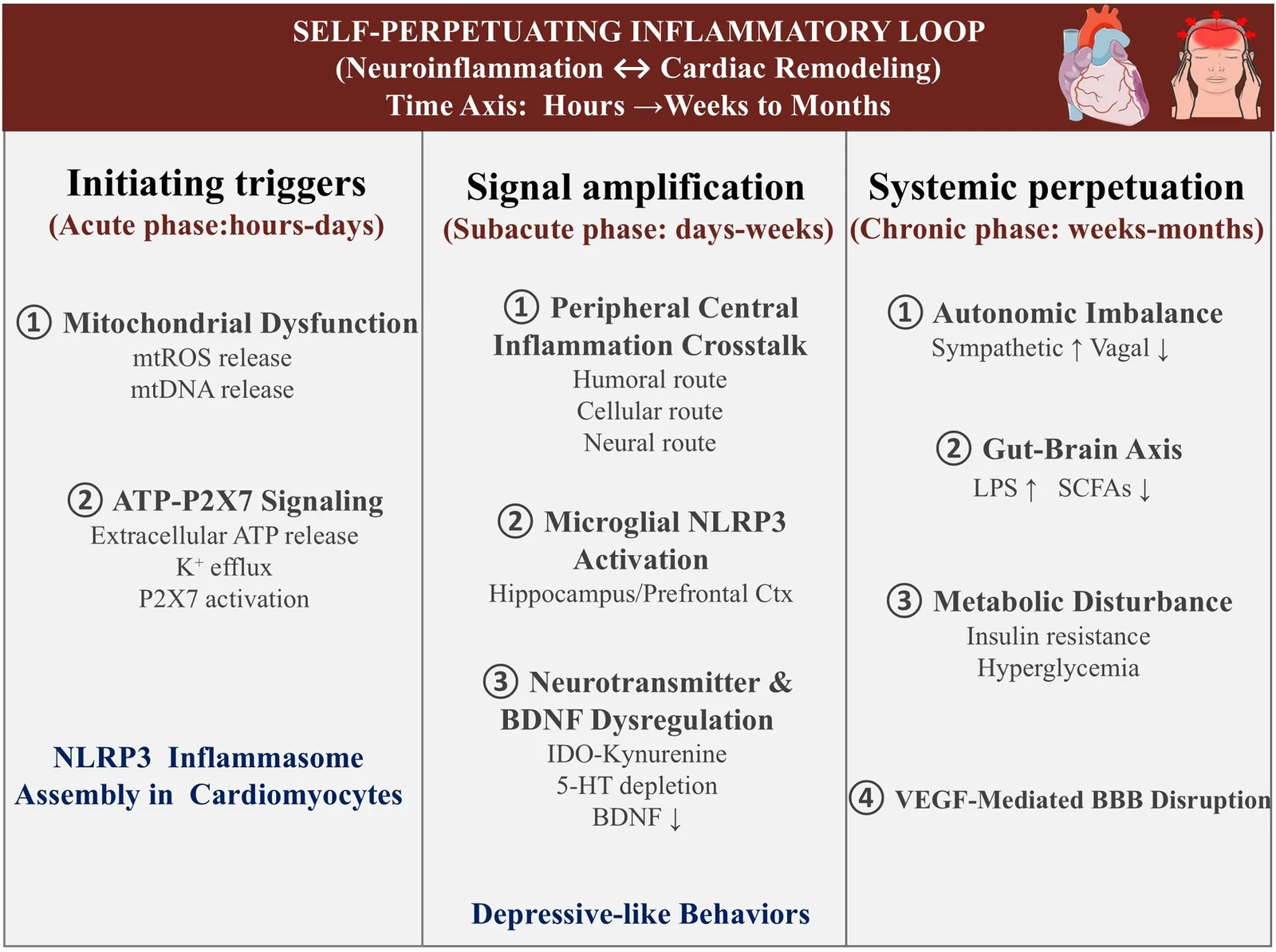
Hierarchical spatiotemporal framework of NLRP3-mediated mechanisms in myocardial infarction-depression comorbidity. The pathological pathways are organized into three temporal layers. Layer 1 (Initiating Triggers, acute phase) involves mitochondrial dysfunction (mtROS/mtDNA) and ATP-P2X7 signaling (ATP/K⁺), which act in parallel as independent upstream signals that converge on NLRP3 inflammasome assembly in cardiomyocytes. Layer 2 (Signal Amplification, subacute phase) encompasses peripheral-central inflammatory crosstalk via humoral, cellular, and neural routes, leading to microglial NLRP3 activation in the hippocampus and prefrontal cortex, which in turn induces neurotransmitter and BDNF dysregulation (IDO-kynurenine pathway, 5-HT depletion, BDNF reduction) and ultimately produces depressive-like behaviors. Layer 3 (Systemic Perpetuation, chronic phase) comprises autonomic imbalance (sympathetic overactivation and vagal withdrawal), gut dysbiosis (elevated LPS and reduced SCFAs), metabolic disturbances (insulin resistance and hyperglycemia), and VEGF-mediated blood-brain barrier disruption, all of which act synergistically to sustain NLRP3 activation. These three layers collectively establish a self-perpetuating inflammatory loop in which neuroinflammation and cardiac remodeling reciprocally exacerbate each other.

### 5.10 Temporal dynamics and dose-response relationships: a critical evidence gap in NLRP3-mediated heart-brain comorbidity

The temporal sequence and dose-response relationship between cardiac NLRP3 activation and subsequent central neuroinflammation remain insufficiently addressed. Establishing these dimensions is essential for distinguishing causal mediation from mere association and identifying rational therapeutic windows.

In single-disease models, NLRP3 activation is relatively well characterized. In myocardial ischemia-reperfusion, inflammasome assembly occurs rapidly after reperfusion, with IL-1β peaking within hours and declining over days (24, 80). NLRP3 inhibition is effective only within a narrow post-reperfusion window, and transcriptomic analysis has revealed time-dependent activation patterns at early reperfusion time points (2, 9). In chronic stress-induced depression models, hippocampal NLRP3 upregulation is detectable after sustained stress and persists for weeks (11, 92). However, in comorbidity models, the temporal relationship between these events has not been mapped. It remains unknown whether central NLRP3 activation represents a direct consequence of peripheral signals reaching the brain within hours, a secondary wave developing over days to weeks, or a parallel process beginning early but persisting chronically.

No study has established a dose-response relationship between cardiac NLRP3 activation magnitude and post-MI depression severity. The Baysak et al. finding is a statistical association, and its observational design precludes causal inferences. Preclinical studies manipulating infarct size or NLRP3 expression levels and correlating these with depressive outcomes are absent. Thus, the causal role of NLRP3 remains inferential. Available evidence allows partial temporal inferences. Minocycline initiated after MI reduced hippocampal NLRP3 expression and improved cardiac function and depressive-like behaviors (83). MI-derived extracellular vesicles induced hippocampal NLRP3 activation and depressive-like behaviors within days (84), and post-MI patients exhibit elevated IL-1β levels in the days following infarction (1). However, these studies do not establish when central NLRP3 activation begins, how its magnitude correlates with infarct size, or whether early inhibition is more effective than delayed intervention. Establishing these temporal and dose-response relationships is essential for distinguishing causal mediation from mere association and for identifying rational therapeutic windows.

## 6 Therapeutic strategies targeting the NLRP3 inflammasome

Given the proposed role of the NLRP3 inflammasome in the comorbidity of myocardial infarction and depression, therapeutic strategies targeting this inflammasome and its associated signaling pathways have attracted increasing research interest. Currently, therapeutic approaches targeting the NLRP3 inflammasome can be broadly divided into two major categories: direct inhibition of NLRP3 inflammasome assembly and activation, and indirect modulation of its upstream signaling pathways. However, most of the evidence discussed below derives from single-disease models, and the translation of these findings to comorbid patients requires further investigation.

### 6.1 NLRP3 inhibitors and targeting of downstream effector molecules

All direct NLRP3 inhibitors described below share a common downstream effect of reducing IL-1β and IL-18 maturation and secretion, although they differ in their molecular targets, binding sites, and pharmacokinetic properties.

MCC950 is the most extensively studied selective small molecule NLRP3 inhibitor. It acts by binding to the NACHT domain of NLRP3, blocking its ATPase activity, thereby inhibiting NLRP3 oligomerization and inflammasome assembly. In animal models of acute myocardial infarction, MCC950 administration effectively reduces hypoxia induced cardiomyocyte injury, attenuates myocardial oxidative stress, apoptosis, and inflammatory response (130). Furthermore, studies have demonstrated that MCC950 inhibits NLRP3 inflammasome mediated pyroptosis and reduces circulating levels of the pyroptosis execution protein gasdermin D. In mouse models of myocardial infarction, MCC950 treatment improves myocardial survival and left ventricular function, including fractional shortening, wall motion score index, and stroke volume, while also reducing histological infarct size (130). MCC950 has also been shown to improve depressive-like behaviors in chronic stress-induced depression models. These findings suggest its potential to simultaneously target myocardial damage and neuroinflammation, making it a promising candidate for treating heart-brain comorbidity.

INF4E or INF200 represents a new generation of non-sulfonylurea NLRP3 inhibitors. Studies have shown that in isolated rat hearts, pretreatment with INF4E at 50 μM 20 min before ischemia reduces infarct size, representing approximately 20 percent reduction, and doubles left ventricular pressure recovery (131). Mechanistic investigations revealed that INF4E inhibits NLRP3 inflammasome formation, activates the RISK survival pathway involving ERK1/2, Akt, and GSK-3β, and improves mitochondrial function (131). Mitochondrial dysfunction is a common pathological mechanism for myocardial ischemia and stress-induced depression. Therefore, INF4E may have dual therapeutic benefits for comorbid conditions, but this needs to be verified in comorbidity models.

OLT1177, also known as dapansutrile is the first NLRP3 inhibitor to enter clinical trials. In a Phase 1b clinical trial involving post myocardial infarction patients with heart failure, OLT1177 administered at 2,000 mg per day for 14 days increased left ventricular ejection fraction and extended exercise duration from 570 to 616 s, with no severe adverse events (132, 133). Whether the anti-inflammatory effect of OLT1177 translates to benefits for patients at high risk of post-myocardial infarction depression remains to be determined, although elevated IL-1β and IL-18 levels are associated with both cardiac dysfunction and depressive symptoms (134).

MRT-8102 is a NEK7 targeting molecular glue degrader developed by Monte Rosa Therapeutics. In Phase 1 clinical trials involving subjects with elevated cardiovascular disease risk, treatment for 4 weeks reduced high sensitivity C reactive protein by 85 percent, with 94 percent of subjects achieving high sensitivity C reactive protein below 2 mg per liter from a median baseline of 6.3 mg per liter. NEK7 degradation reached 80–90 percent, with mild to moderate adverse events and no evidence of increased infection risk. NEK7 is an important regulatory factor for the assembly of the NLRP3 inflammasome. Targeting this protein may provide a more selective strategy for inhibiting NLRP3, and at the same time act on the pathological processes of the heart and nervous system, thereby resolving the bidirectional relationship of myocardial infarction and depression comorbidity. Selnoflast is an oral NLRP3 inhibitor that has shown safety and target engagement in a Phase 1b trial in ulcerative colitis, but its efficacy in coronary artery disease or post-MI depression remains to be evaluated (135).

VX-765, a caspase-1 inhibitor, blocks the maturation and secretion of interleukin 1β and interleukin 18 by inhibiting caspase-1 activation. In a mouse model of acute myocardial ischemia, VX-765 effectively reduces hypoxia induced cardiomyocyte injury and attenuates myocardial redox imbalance, apoptosis, and inflammation. Both VX-765 and MCC950 have been confirmed to improve myocardial injury by inhibiting NLRP3 inflammasome mediated pyroptosis (130). By reducing systemic inflammation, selnoflast may also alleviate the central inflammatory response that leads to post-myocardial infarction depression. However, this needs to be directly verified in patients with comorbidities in Table 3.

**Table 3:** Pharmacological agents targeting the NLRP3 inflammasome: preclinical and clinical evidence relevant to MI-depression comorbidity.

Drug	Category	Primary mechanism of action	Study type	Evidence in MI models	Evidence in depression models	Clinical development stage	Refs.
MCC950	Direct NLRP3 inhibitor	Binds NLRP3 NACHT domain, inhibits ATPase activity and oligomerization	Comorbidity/MI model	Reduces infarct size, improves LV function, lowers GSDMD levels	Improves chronic stress-induced depressive-like behaviors	Preclinical	(130)
INF4E/INF200	Direct NLRP3 inhibitor	Inhibits NLRP3 inflammasome assembly	MI model	Reduces infarct size, activates RISK pathway, improves mitochondrial function	Lacks evidence in comorbidity models	Preclinical	(131)
OLT1177 (Dapansutrile)	Direct NLRP3 inhibitor	Inhibits NLRP3 ATPase activity	Clinical (MI only)/Preclinical	Phase Ib trial (post-MI HF patients) shows improved LVEF and exercise duration	Preclinical studies suggest antidepressant effects	Phase Ib	(133)
BGE-102	Direct NLRP3 inhibitor	Brain-penetrant NLRP3 inhibitor	Preclinical (MI model/depression model)	Preclinical studies show reduced infarct size	Preclinical studies suggest antidepressant effects	Phase I	–
MRT-8102	NEK7-targeted degrader	Degrades NEK7, prevents NLRP3 inflammasome assembly	Preclinical (MI model/depression model)	Preclinical studies show cardioprotective effects	Preclinical studies show neuroprotective effects	Phase I	–
Selnoflast	Direct NLRP3 inhibitor	Oral NLRP3 inhibitor	MI model	Preclinical studies show anti-atherosclerotic effects	Lacks evidence in MI models	Phase Ib (ulcerative colitis)	(135)
VX-765	Caspase-1 inhibitor	Inhibits caspase-1, blocks IL-1β/IL-18 maturation	Comorbidity/MI model	Reduces myocardial infarct size, inhibits cardiomyocyte pyroptosis	Improves stress-induced depressive behaviors	Preclinical	(130)
Colchicine	Indirect NLRP3 inhibitor	Inhibits microtubule polymerization, interferes with inflammasome assembly	Clinical (MI only)	Phase III trials (COLCOT, LoDoCo2) show reduced MACE	Lacks clinical evidence	Phase III	(136)
Metformin	Indirect NLRP3 inhibitor	Activates AMPK, inhibits NF-κB nuclear translocation, enhances autophagy	MI model/Clinical observation	Improves diabetic cardiomyopathy, inhibits cardiac fibrosis	Clinical observations show reduced depression risk	Preclinical and clinical observation	(137)
Statins	Indirect NLRP3 inhibitor	Inhibits mevalonate pathway, downregulates NLRP3 expression	Clinical (MI)/Cohort (depression)	Large clinical trials confirm cardiovascular benefits	Cohort studies show reduced depression risk	Approved	(138)
Paeoniflorin	Natural product	Activates SIRT1, promotes autophagy, inhibits NF-κB	Comorbidity/MI model	Attenuates myocardial ischemia-reperfusion injury, improves cardiac function	Improves CUMS-induced depressive behaviors	Preclinical	(59, 61, 62)
Dapagliflozin	SGLT2 inhibitor	Upregulates SIRT1, inhibits NLRP3 inflammasome activation	Comorbidity model	Improves post-MI ventricular remodeling and cardiac function	Improves depressive-like behaviors in post-MI comorbid depression rats	Approved (for heart failure)	(58)

MI, myocardial infarction; LVEF, left ventricular ejection fraction; RISK, reperfusion injury salvage kinase; MACE, major adverse cardiovascular events; AMPK, AMP-activated protein kinase; NF-κB, nuclear factor-κB; CUMS, chronic unpredictable mild stress; SGLT2, sodium-glucose cotransporter 2. Study type indicates whether the evidence derives from comorbidity-specific models/patients or single-disease models. Comorbidity refers to studies that simultaneously investigated both MI and depression outcomes; MI model or depression model refers to single-disease studies providing indirect evidence relevant to the comorbidity context.

### 6.2 Indirect inhibitors

Colchicine, a microtubule inhibitor, blocks NLRP3 inflammasome assembly by inhibiting microtubule polymerization. In the COLCOT and LoDoCo2 randomized controlled trials, low dose colchicine at 0.5 mg per day significantly reduced the incidence of major adverse cardiovascular events in post myocardial infarction patients, with relative risk reductions (136). Colchicine may also exert an antidepressant effect through its anti-inflammatory properties, as systemic inflammation is a common driving factor for both atherosclerosis and depression.

Metformin, a classic antidiabetic drug, activates the AMPK signaling pathway, inhibits NF κB nuclear translocation, and reduces the expression of NLRP3, IL-1β, and IL-18 (137). In animal models of diabetic cardiomyopathy, metformin attenuates fibrosis and protects cardiac function by inhibiting the NLRP3 inflammasome and enhancing autophagy (137). It is worth noting that metformin has also been proven to improve depressive-like behaviors in animal models and can reduce the risk of depression in diabetic patients, demonstrating its potential as a dual treatment drug for comorbid cardiovascular and cerebrovascular diseases.

Statins including atorvastatin and simvastatin, as lipid lowering agents, alleviate oxidized low-density lipoprotein induced endothelial injury and diabetic cardiomyopathy, with mechanisms involving inhibition of NLRP3 activation. Statins also reduce mevalonate pathway flux, thereby disrupting trained immunity in monocytes and macrophages (138). In addition to the cardiovascular benefits, large-scale cohort studies have shown that the use of statins is associated with a reduced risk of depression, suggesting that their anti-inflammatory and immunomodulatory effects may extend to the central nervous system.

In a mouse model of chronic unpredictable mild stress-induced depression, paeoniflorin exerts antidepressant effects through multiple mechanisms. Specifically, it activates the SIRT1 signaling pathway, inhibits NF-κB nuclear translocation, and downregulates NLRP3, caspase-1, and gasdermin D N-terminal expression (138). It is worth noting that paeoniflorin has also been proven to protect against myocardial ischemia-reperfusion injury in animal models, suggesting that it may simultaneously target both cardiac and neurological pathological processes. Other traditional Chinese medicine active ingredients such as resveratrol, salvianolic acid B, and berberine also inhibit NLRP3 inflammasome activation through multi target mechanisms, demonstrating potential in both cardiovascular protection and antidepressant effects (138).

### 6.3 Comparative analysis of therapeutic strategies

The therapeutic agents discussed above can be classified into three mechanistic categories, direct NLRP3 inhibitors, upstream pathway modulators, and pleiotropic natural products (Table 4**)**. These categories differ substantially in target specificity, brain penetrance, safety profiles, and translational readiness.

**Table 4:** Summary of key experimental and clinical studies on NLRP3-targeted interventions in MI-depression comorbidity.

Study	Disease model	Study type	Intervention	Molecular target	Principal findings	Translational significance	Refs.
Wang et al.	Rat: MI + CUMS	Comorbidity model	Dapagliflozin	SIRT1/NLRP3	Improved cardiac function and depressive-like behaviors; reduced pyroptosis via SIRT1 upregulation	SGLT2 inhibitors may have dual benefits for post-MI depression	(58)
Su et al.	Mouse: CUMS	Comorbidity/MI model	Paeoniflorin	NLRP3 inflammasome	Reduced NLRP3/ASC/caspase-1/IL-1β expression; restored mitochondrial function via autophagy enhancement	Natural products targeting mitophagy-NLRP3 axis warrant clinical investigation	(59)
Ye et al.	Mouse: acute MI	MI model	MCC950, VX-765	NLRP3, caspase-1	Reduced infarct size; attenuated oxidative stress, apoptosis, and inflammation	Direct NLRP3/caspase-1 inhibitors show cardioprotective potential	(130)
Lu et al.	Rat: I/R injury	MI model	Magnetic vagus nerve stimulation	M_2_AChR/OGDHL/ROS axis	Reduced infarct size; inhibited NLRP3-mediated pyroptosis; improved mitochondrial function	Non-invasive vagus stimulation may offer dual cardiac and antidepressant effects	(97)
Wang et al.	Rat: chronic MI	Comorbidity model	Fecal microbiota transplantation	Gut microbiota-NLRP3 axis	Transferred depressive-like behaviors; altered gut microbiota composition	Gut microbiota modulation represents a novel therapeutic strategy for post-MI depression	(98)
Sun et al.	Mouse: cardiac arrest	Cardiac arrest model (indirect evidence)	Sodium butyrate	TLR4/MyD88/NF-κB pathway	Improved neurological outcomes; inhibited M1 microglial polarization and neuroinflammation	SCFAs or SCFA-producing probiotics may alleviate post-MI neuroinflammation	(103)
Wang et al.	Rat: MI	Comorbidity model	Minocycline	Microglial NLRP3 inflammasome	Reduced hippocampal NLRP3 expression; improved cardiac function and depressive-like behaviors	Microglia-targeted anti-inflammatory drugs can simultaneously improve cardiac and depressive outcomes	(83)
Baysak et al.	Human: STEMI patients	Clinical (post-MI depression)	Observational (plasma NLRP3)	NLRP3	Plasma NLRP3 levels were independently associated with depression occurrence (OR = 1.72)	NLRP3 may serve as a biomarker for post-MI depression risk	(33)
Wohlford et al.	Human: post-MI HF (Phase 1b)	Clinical (MI only)	OLT1177 (Dapansutrile)	NLRP3 ATPase	Improved LVEF; extended exercise duration; no severe adverse events	First NLRP3 inhibitor with clinical safety data in post-MI patients; antidepressant effects require testing	(133)
Wenzel et al.	*In vitro*: THP-1, HL-60 cells	*In vitro* model	SCFA mixture	NLRP3 inflammasome	Reduced IL-1β/TNF-α secretion; inhibited respiratory burst and ROS production	SCFAs directly modulate immune cell NLRP3 activation	(104)

MI, myocardial infarction; CUMS, chronic unpredictable mild stress; I/R, ischemia-reperfusion; SCFA, short-chain fatty acid; LVEF, left ventricular ejection fraction; HF, heart failure; OR, odds ratio; ROS, reactive oxygen species. Study Type indicates whether the study employed comorbidity-specific models/patients or single-disease models, allowing readers to distinguish direct evidence from comorbidity systems from indirect evidence derived from single-disease studies.

Direct NLRP3 inhibitors offer high target specificity. OLT1177 and BGE-102 have demonstrated clinical safety and brain penetrance, properties that make them attractive for targeting both peripheral and central inflammation in MI-depression comorbidity. However, their inhibition of the IL-1β/IL-18 pathway raises theoretical immunosuppression concerns, though no significant safety signals have emerged to date. MCC950 and INF4E remain in preclinical stages with limited long-term safety data. Upstream modulators benefit from established clinical safety records through decades of use, facilitating rapid repurposing. Metformin and statins additionally address underlying metabolic risk factors that contribute to both MI and depression. However, their NLRP3 inhibition is indirect and pleiotropic, making it difficult to attribute therapeutic benefits specifically to NLRP3 modulation, and clinical evidence for antidepressant effects remains inconsistent. Colchicine has robust cardiovascular outcome data but its antidepressant potential has not been systematically evaluated, and gastrointestinal intolerance limits tolerability.

Natural products exhibit multi-target actions that may benefit complex comorbidities, simultaneously modulating NLRP3 activation, oxidative stress, and mitochondrial function. Their clinical translation, however, is constrained by poor oral bioavailability, lack of standardized formulations, and insufficient human pharmacokinetic and safety data. The absence of large-scale trials for natural products in this comorbidity represents a substantial evidence gap. Dapagliflozin, an SGLT2 inhibitor, has well-established cardiovascular and renal benefits from large outcomes trials and emerging preclinical evidence for antidepressant effects. Its favorable safety profile and once-daily oral dosing make it a strong candidate for clinical investigation, though its antidepressant effects currently rest on a single animal study.

Several common translational limitations apply across all categories. Preclinical studies have been conducted almost exclusively in young adult male animals, with no systematic evaluation of sex differences, age effects, or prevalent comorbidities. The optimal timing of intervention relative to MI onset remains undefined, despite NLRP3 serving different functions across acute injury, inflammatory amplification, and chronic remodeling phases. Drug-drug interactions with standard post-MI medications have not been systematically examined. Finally, no validated biomarker exists to identify patients most likely to respond to NLRP3-targeted therapies, a prerequisite for patient stratification in clinical trials.

### 6.4 Critical evidence gaps in translating from single-disease to comorbidity

Although NLRP3 inhibition holds therapeutic promise for MI and depression individually, no prospective clinical trial has tested any NLRP3-targeted agent specifically in patients with comorbid MI and depression. Before NLRP3-targeted therapies can be rationally evaluated in this specific patient group, at least five critical evidence gaps must be addressed. First, the predictive value of baseline NLRP3-related biomarkers for antidepressant efficacy in post-MI patients remains unknown. Second, the impact of NLRP3 inhibition on ventricular remodeling and repair must be characterized across different post-MI time windows, as NLRP3 plays dual roles in both injury and repair. Third, pharmacokinetic and drug-drug interaction profiles in the context of polypharmacy (antiplatelets, statins, β-blockers, antidepressants) have not been systematically evaluated. Fourth, the optimal timing of intervention relative to MI onset is undefined, given that NLRP3 serves different functions across acute, subacute, and chronic phases. Fifth, the long-term safety of chronic NLRP3 inhibition in frail, multimorbid post-MI patients requires dedicated investigation. These five gaps must be addressed before NLRP3-targeted therapies can be rationally evaluated in the comorbid population.

## 7 Discussion

Recent studies have advanced the understanding of NLRP3 inflammasome in myocardial infarction (MI) and its related brain complications from multiple levels. A study combining single-cell RNA sequencing (scRNA-seq), single-nucleus RNA sequencing (snRNA-seq), and spatial transcriptomics revealed that pro-inflammatory macrophages in post-MI cardiac tissues specifically upregulate TIM-3, accompanied by enhanced NLRP3 inflammasome activity. Mechanistically, cardiomyocyte-derived HMGB-1 drives macrophage pro-inflammatory polarization and NLRP3 activation through the TIM-3/HMGB-1 axis. These findings establish an immune checkpoint regulatory mechanism of post-MI inflammation at single-cell and spatial resolution (139). Another study integrating four MI patient bulk RNA-seq datasets with one snRNA-seq dataset identified NLRP3, IL-1β, and TLR4 as key biomarkers of PANoptosis (pyroptosis-apoptosis-necroptosis) in MI, with single-cell analysis confirming predominant expression of these genes in monocytes/macrophages (140). At the heart-brain interface, a study demonstrated that downregulation of cardiac SLC22A3 in post-MI heart failure mice leads to peripheral histamine accumulation, which enters the hippocampus through the disrupted blood-brain barrier and activates microglial H1R/NLRP3 pathway, driving neuroinflammation and cognitive impairment, delineating a complete cardiac-neuroinflammatory axis that provides important supporting evidence for understanding post-MI central nervous system complications, including depression (141). In comorbid MI-depression models, transcriptomic and proteomic studies showed that Shuangxinfang simultaneously improves cardiac function and depressive-like behaviors by inhibiting the S100A9/NLRP3 pathway and ameliorating mitochondrial energy metabolism (142), while Shexiang Baoxin Pills exert dual cardioprotective and antidepressant effects by downregulating hippocampal NLRP3, ASC, Caspase-1, and GSDMD expression to inhibit pyroptosis (143).

Several 2026 studies have further strengthened the link between NLRP3, MI, and depression. In comorbid MI–depression models, Shuangxinfang ameliorated post-MI depression by targeting S100A9/NLRP3-mediated mitochondrial dysfunction, while modulation of the M1/TNF-α/TNFR1/NF-κB axis offered an additional therapeutic route (144, 145). A 2026 NHANES study linked systemic immune-inflammation and platelet parameters to depressive symptoms in MI survivors (146). In addition, a 2026 systematic review and meta-analysis confirmed NLRP3 inflammasome priming and activation in depression (147). Collectively, these findings provide multi-omics and single-cell evidence supporting NLRP3 as a molecular bridge in MI-depression comorbidity and highlight the therapeutic potential of targeting NLRP3 and its associated immune-neural pathways.

The proposed framework suggests that NLRP3 acts as a self-sustaining inflammatory hub linking cardiac injury and central neuroinflammation. This integrated view reframes the comorbidity as a self-perpetuating loop and implies that therapeutic strategies may need to be stage-dependent. However, establishing causality will require longitudinal studies with repeated measurements, cell-type-specific conditional knockout approaches, and comparative trials administering NLRP3 inhibitors at different post-MI time points. Changing clinical practice would require prospective trials showing that NLRP3-related biomarkers can identify post-MI patients at risk of depression or treatment resistance, and that NLRP3 inhibition improves both depressive and cardiovascular outcomes when added to standard care, while the optimal timing, long-term safety in multimorbid patients, and absence of harm to cardiac repair would also need to be established; until then, NLRP3-targeted therapy should remain investigational, and the most realistic near-term advance would be biomarker-guided stratification in clinical trials rather than routine clinical use.

### 7.1 Limitations

This review has several limitations. It is a narrative review rather than a systematic review or meta-analysis, and no formal protocol registration, risk-of-bias assessment, or quantitative synthesis was performed. A major limitation of the current evidence base is the scarcity of direct evidence from comorbidity-specific experimental systems. As noted in Section 4, the vast majority of studies reviewed derive from single-disease models of either MI or depression, providing only indirect support for NLRP3-mediated heart–brain crosstalk. Several pathways, including the ATP–P2X7 axis, VEGF-mediated vascular permeability, and the gut–brain axis, remain supported mainly by indirect or preclinical evidence. Most preclinical studies were conducted in young adult male animals, and sex differences, age effects, and comorbidities were rarely evaluated. The temporal dynamics, dose–response relationships, and optimal therapeutic window of NLRP3 activation in comorbidity remain undefined. Finally, no clinical trial has yet tested NLRP3-targeted therapy specifically in patients with comorbid MI and depression, so the clinical implications proposed here remain hypothetical and require future validation.

### 7.2 Knowledge gaps

Several knowledge gaps remain. The causal role of NLRP3 in MI–depression comorbidity has not been established, the temporal sequence and dose–response relationship of NLRP3 activation across the heart and brain are unknown, and the optimal therapeutic window is undefined. Cell-type-specific contributions and the roles of several pathways, including the ATP–P2X7 axis, VEGF-mediated vascular permeability, and the gut–brain axis, remain unresolved. Validated biomarkers for predicting post-MI depression risk or treatment response are lacking, no clinical trial has tested NLRP3-targeted therapy in comorbid patients, and the long-term safety of chronic NLRP3 inhibition in frail, multimorbid patients remains unknown. Addressing these gaps will require longitudinal comorbidity-specific models, cell-type-specific genetic tools, multi-omics approaches, and prospective biomarker-stratified clinical trials.

### 7.3 Future directions

Future studies should use comorbidity-specific models that simultaneously assess cardiac and depressive outcomes, with longitudinal sampling across acute, subacute, and chronic phases to map NLRP3 activation over time. Cell-type-specific conditional knockout and rescue approaches are needed to define the contributions of cardiomyocytes, macrophages, microglia, astrocytes, and endothelial cells. Multi-omics technologies, including single-cell RNA sequencing, single-cell ATAC sequencing, spatial transcriptomics, proteomics, and metabolomics, should be applied to identify key cell subsets and signaling networks across different pathological stages. Dose–response studies and prospective clinical cohorts with longitudinal biospecimens and biobanks are required to validate NLRP3-related biomarkers and define the optimal therapeutic window. Randomized controlled trials should test whether NLRP3 inhibitors improve both depressive and cardiovascular outcomes in comorbid patients, and long-term safety in frail, multimorbid patients must be evaluated. Multi-target combination strategies, including NLRP3 inhibition with antidepressants, exercise, or microbiota-based interventions, should also be explored. Finally, development of highly selective and low-toxicity NLRP3-targeting drugs is essential; based on structural biology information of NLRP3 inflammasome assembly, next-generation specific inhibitors or degraders can be developed, and natural product-derived NLRP3 inhibitors can be explored as lead compounds with optimized pharmacokinetic and safety profiles.

## 8 Conclusion

This review summarizes the NLRP3 inflammasome as a potential molecular bridge between MI and depression. Current evidence supports its involvement in both cardiac injury and depressive neuroinflammation through multiple interconnected pathways, but most evidence derives from single-disease models, and direct comorbidity-specific evidence remains limited. NLRP3 should therefore be regarded as a promising integrative framework and therapeutic candidate rather than a proven causal mediator. Future comorbidity-specific studies and clinical trials are needed to establish causality and evaluate whether NLRP3-targeted therapy can improve both depressive and cardiovascular outcomes.
